# Correction: Imaging neuropeptide release at synapses with a genetically engineered reporter

**DOI:** 10.7554/eLife.54328

**Published:** 2019-12-11

**Authors:** Keke Ding, Yifu Han, Taylor W Seid, Christopher Buser, Tomomi Karigo, Shishuo Zhang, Dion K Dickman, David J Anderson

Ding K, Han Y, Seid TW, Buser C, Karigo T, Zhang S, Dickman DK, Anderson DJ. 2019. Imaging neuropeptide release at synapses with a genetically engineered reporter. *eLife* **8**:e46421. doi: 10.7554/eLife.46421.

Published 26, June 2019

We recently discovered an error in Figure 1—figure supplement 3, in which the images used in panel B were accidentally duplicated from panel D and mislabeled. This occurred inadvertently during conversion of figure file formats and subsequent figure rearrangement. The figure has been corrected so that panel B now contains the originally intended images. The correction does not affect or change any of the conclusions of the manuscript. We apologize for the mistake and any inconvenience or confusion it may have caused.

The Corrected Figure 1—figure supplement 3 is shown here:

**Figure 1:**
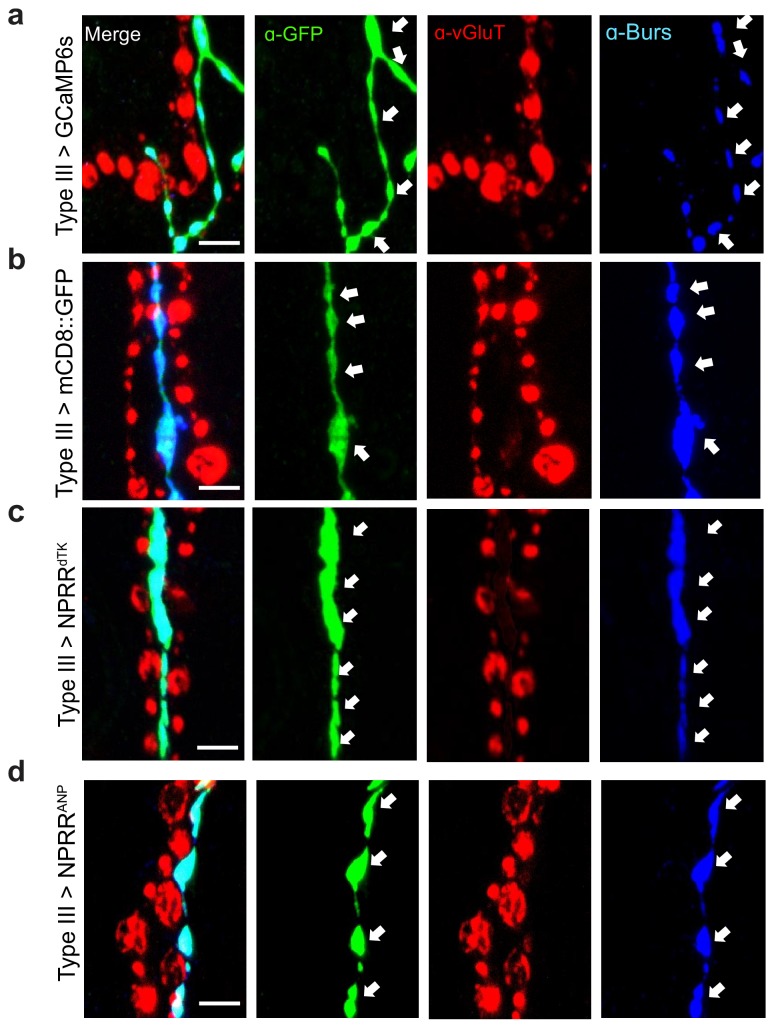


The originally published Figure 1—figure supplement 3 is also shown for reference:

**Figure 2:**
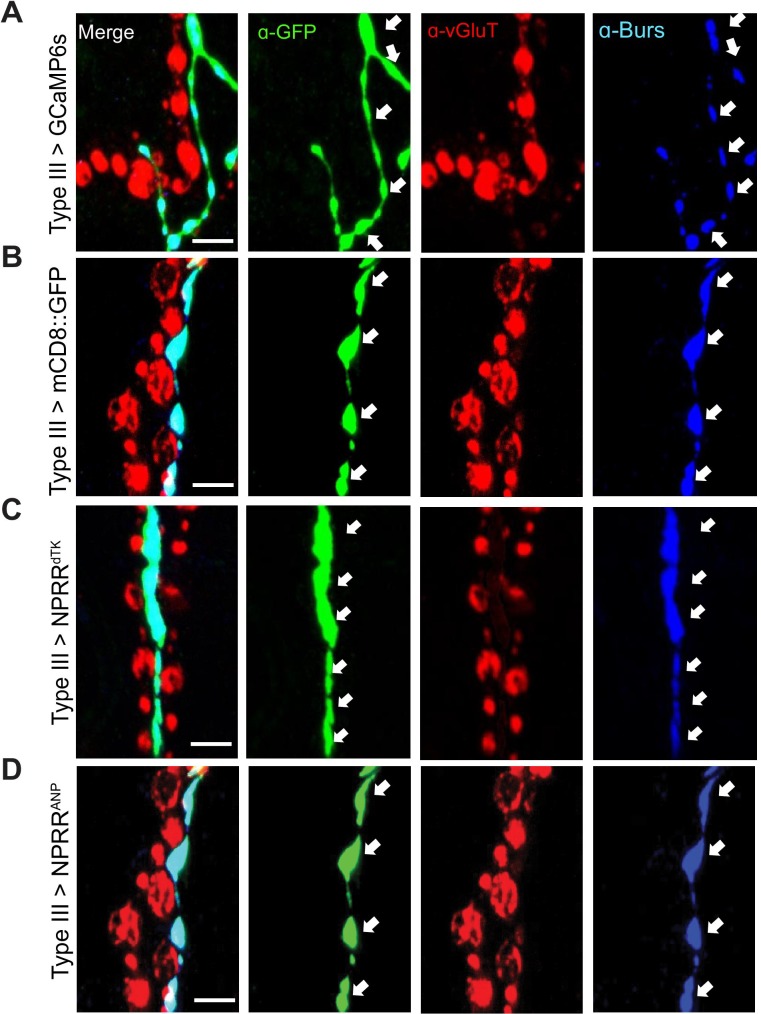


The article has been corrected accordingly.

