# Supplementary figures and images for: Imaging neuropeptide release at synapses with a genetically engineered reporter
Source: eLife. 2019 Jun 26;8:e46421. doi: 10.7554/eLife.46421 (PMC6609332; doi:10.7554/eLife.46421)

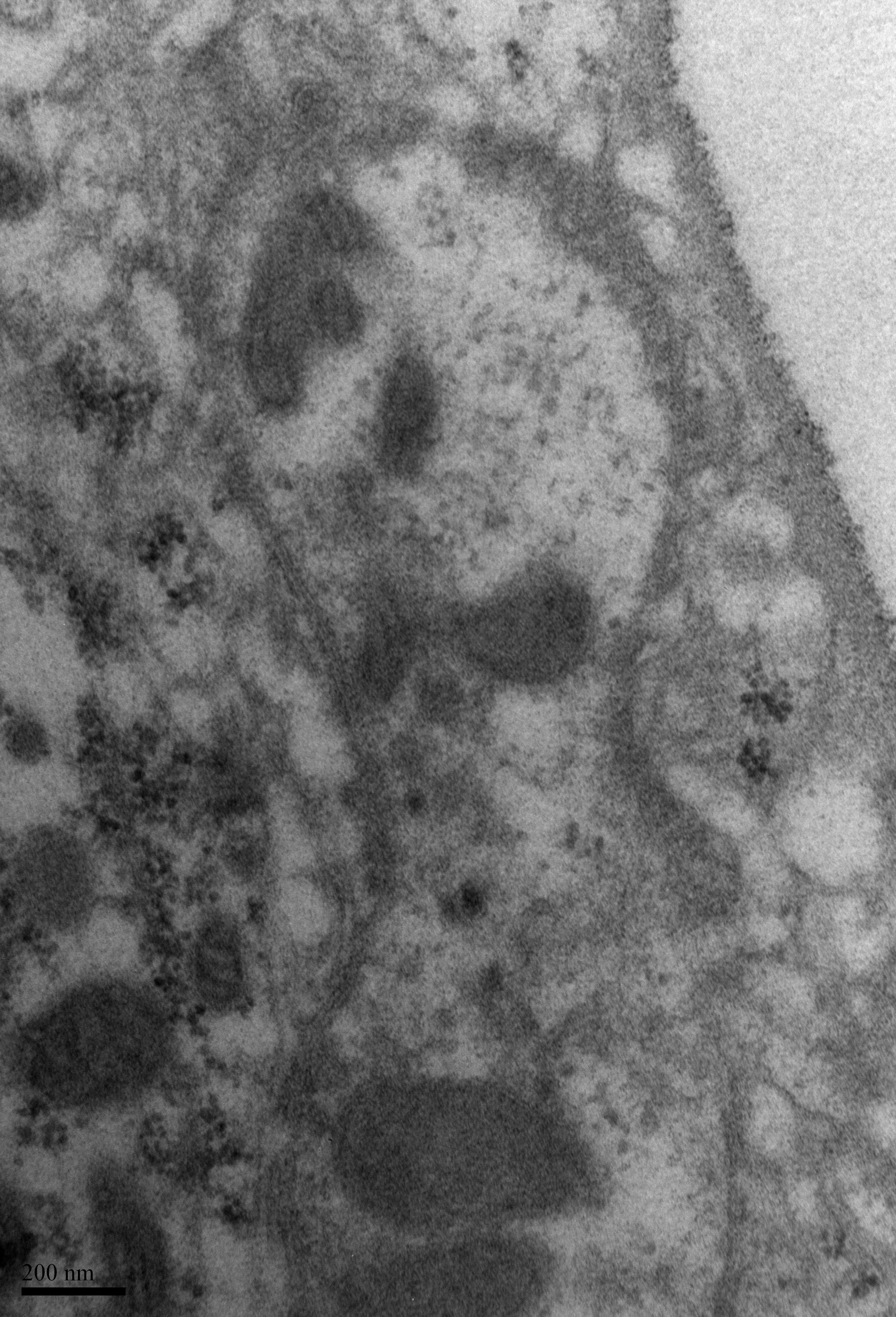

Supplement: Figure 1—source data 2. [file elife-46421-fig1-data2.zip › EM_no_driver_ctrl/003E-01A-bx2D2-001.jpg]

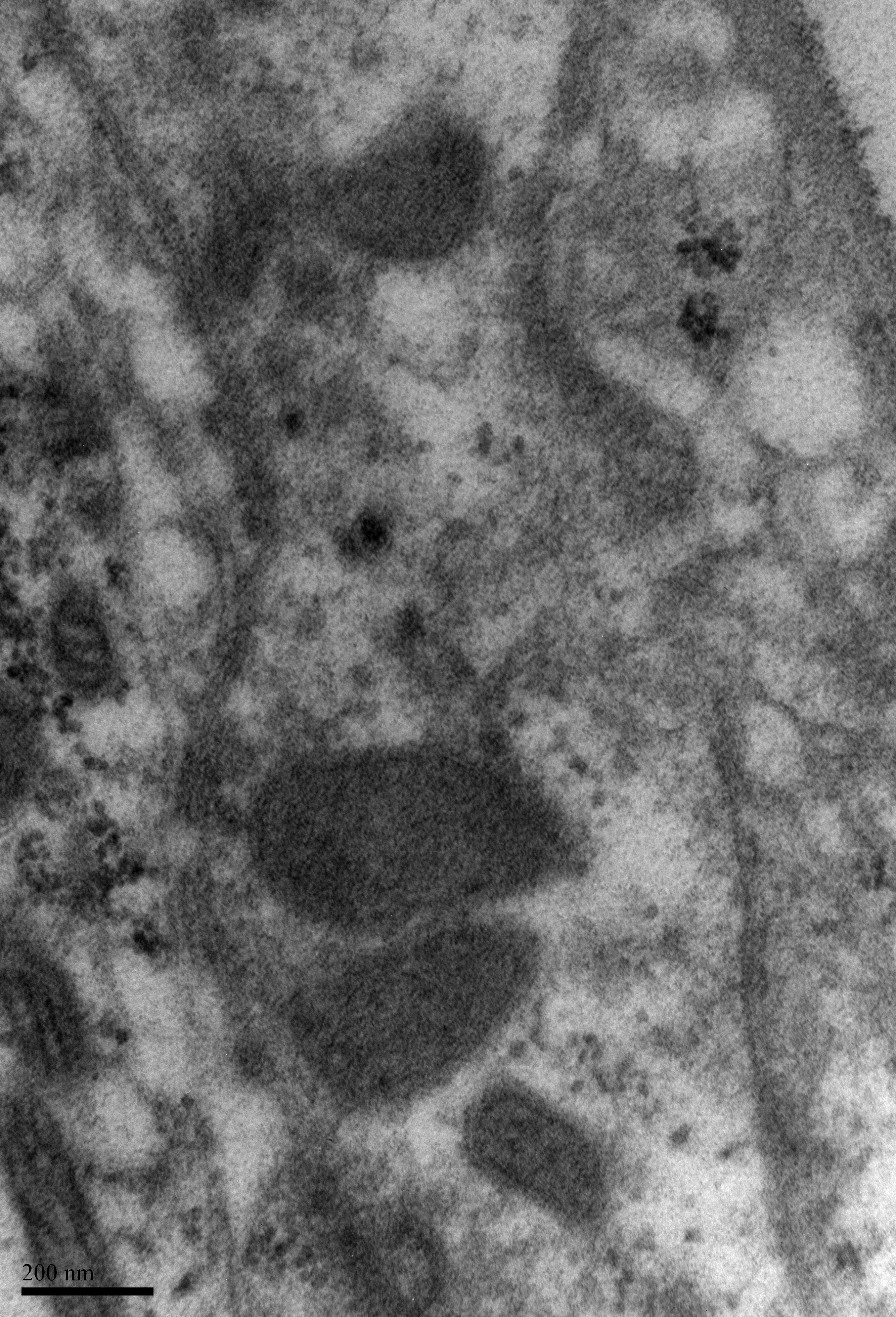

Supplement: Figure 1—source data 2. [file elife-46421-fig1-data2.zip › EM_no_driver_ctrl/003E-01A-bx2D2-002.jpg]

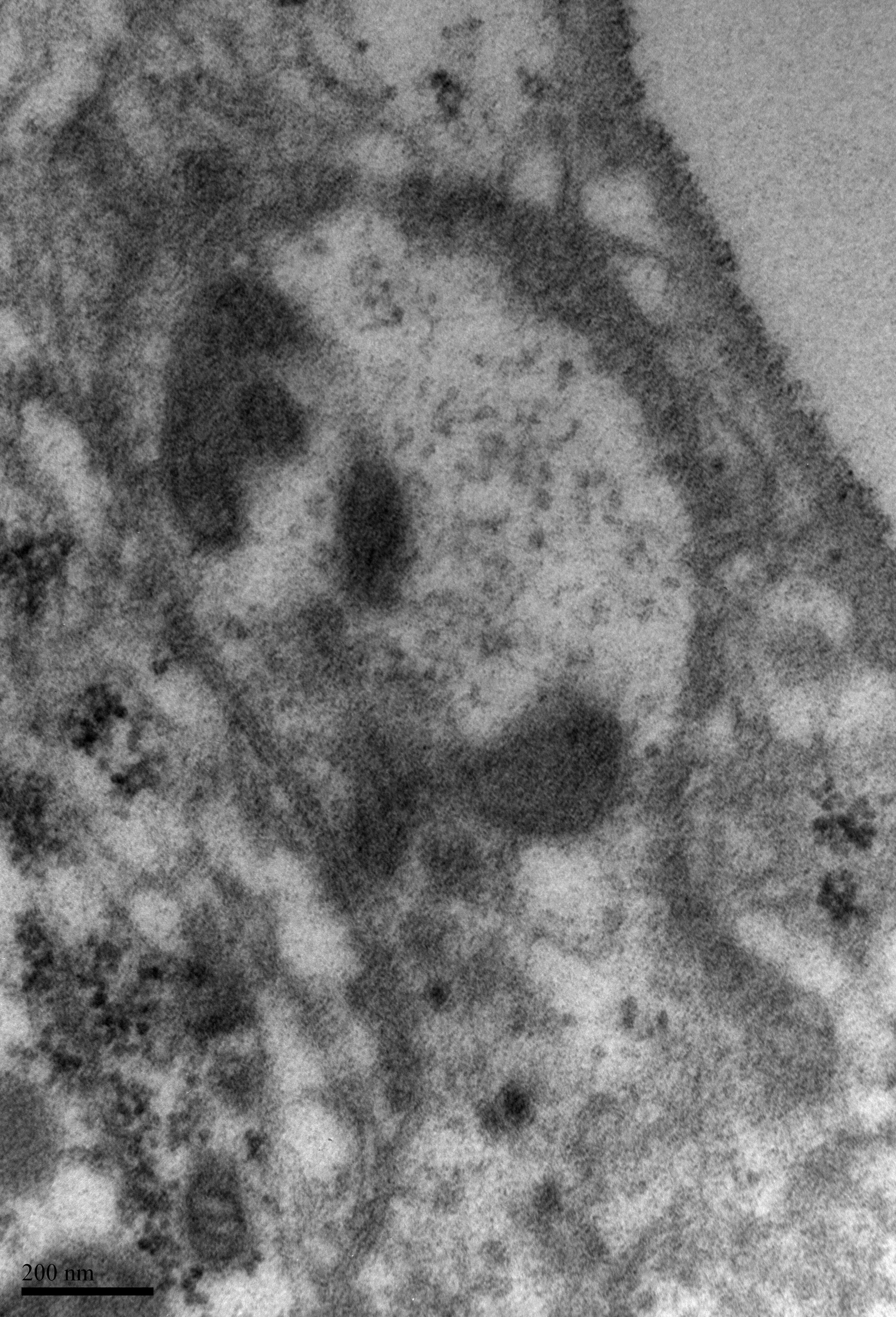

Supplement: Figure 1—source data 2. [file elife-46421-fig1-data2.zip › EM_no_driver_ctrl/003E-01A-bx2D2-003.jpg]

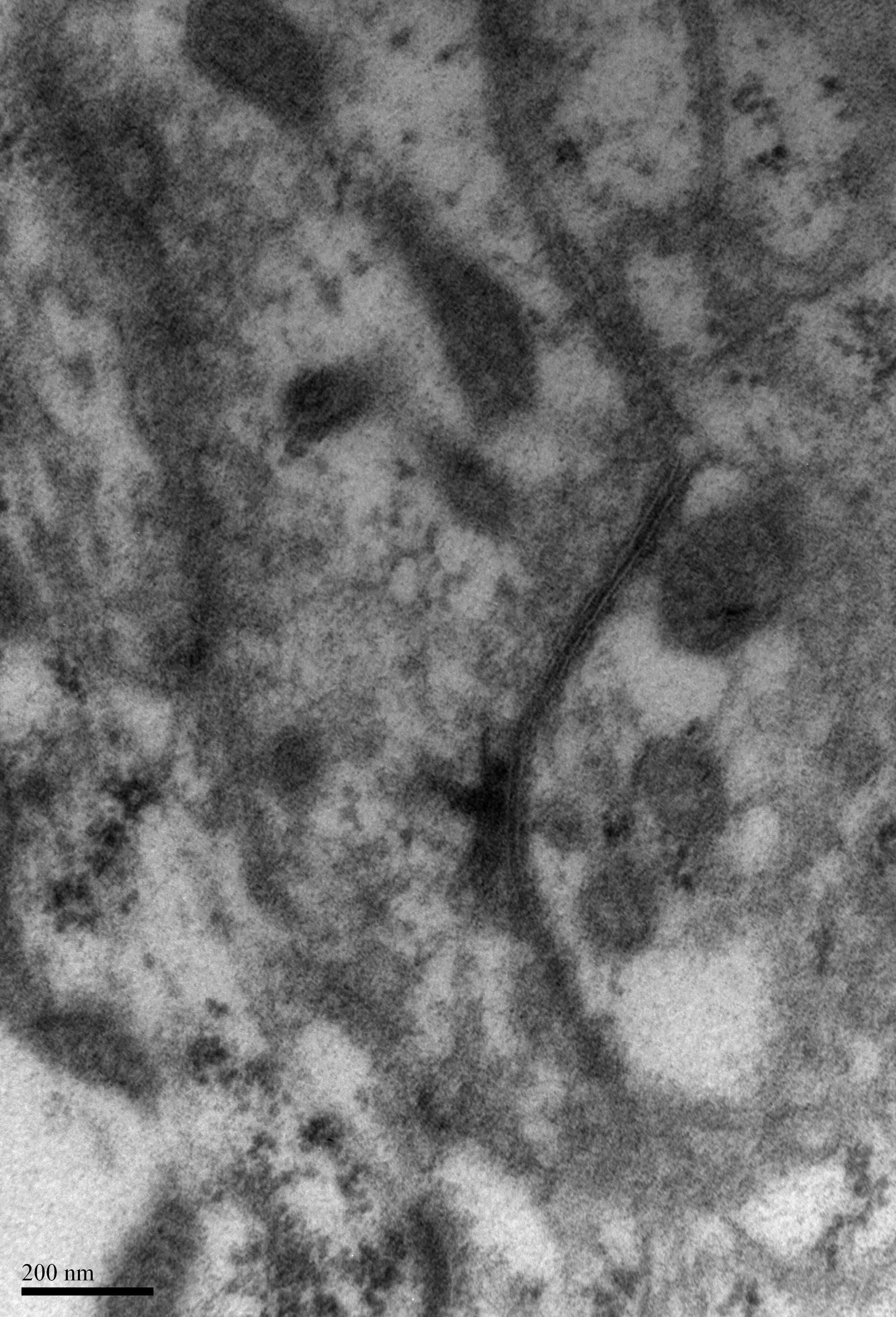

Supplement: Figure 1—source data 2. [file elife-46421-fig1-data2.zip › EM_no_driver_ctrl/003E-01A-bx2D2-004.jpg]

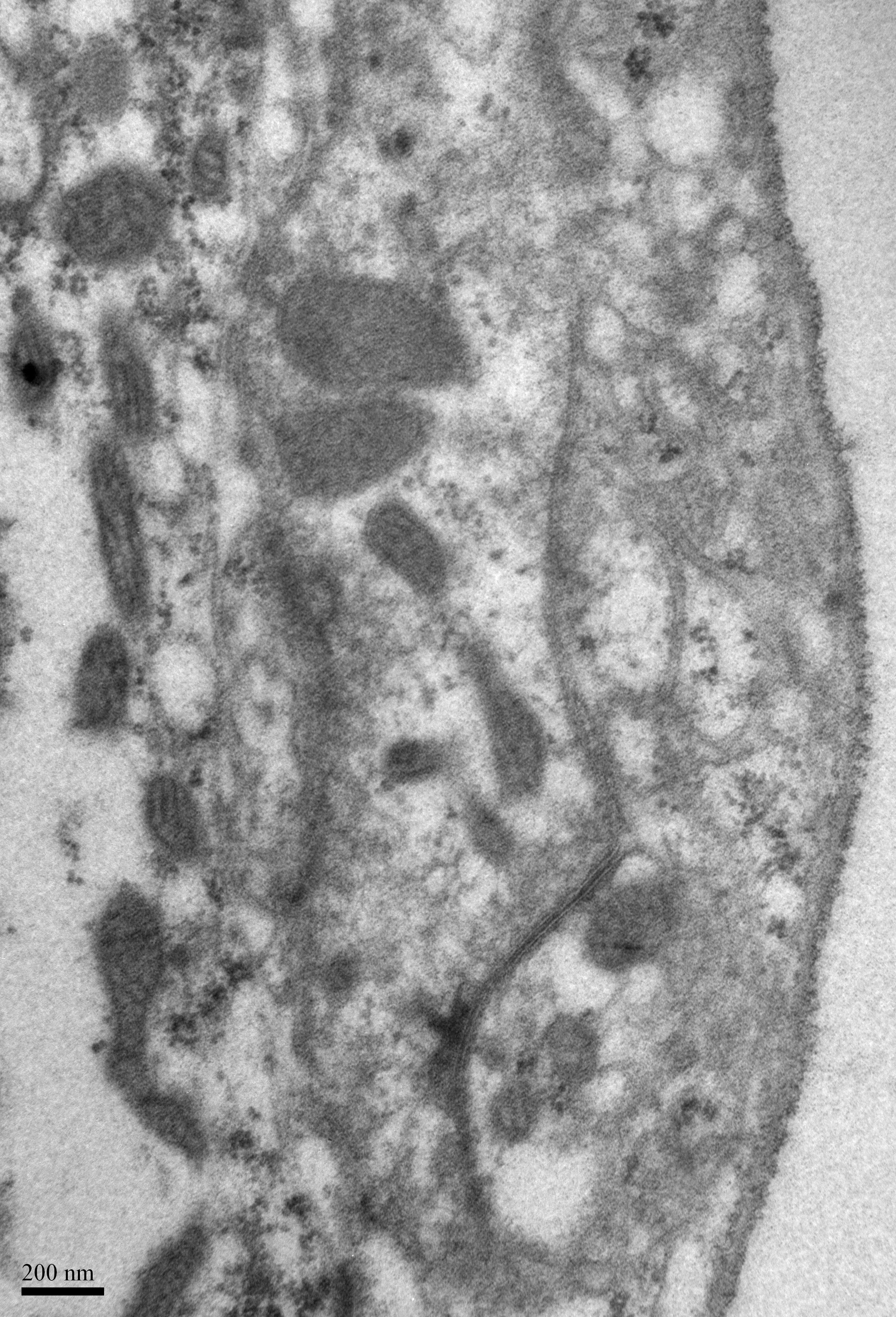

Supplement: Figure 1—source data 2. [file elife-46421-fig1-data2.zip › EM_no_driver_ctrl/003E-01A-bx2D2-005.jpg]

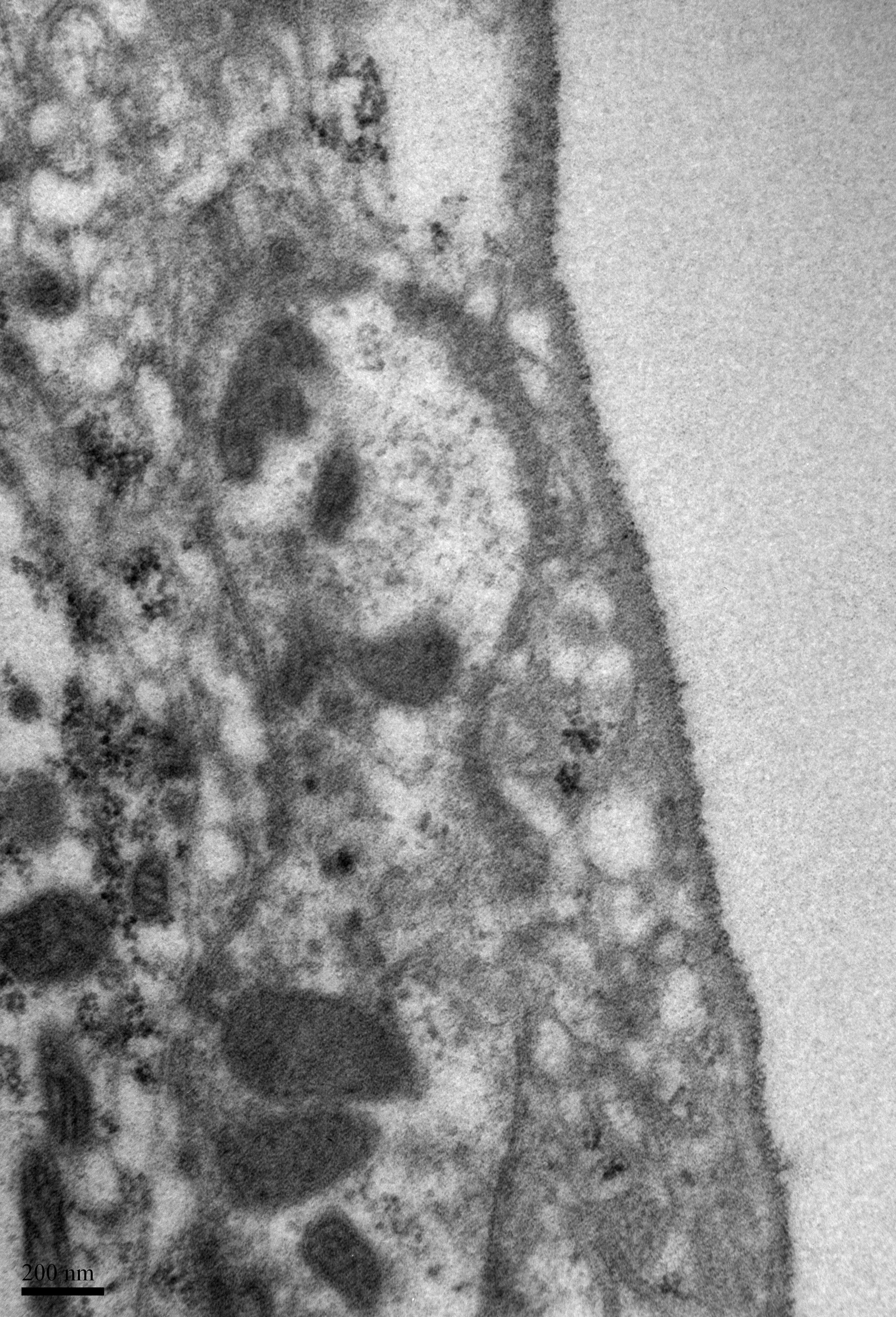

Supplement: Figure 1—source data 2. [file elife-46421-fig1-data2.zip › EM_no_driver_ctrl/003E-01A-bx2D2-006.jpg]

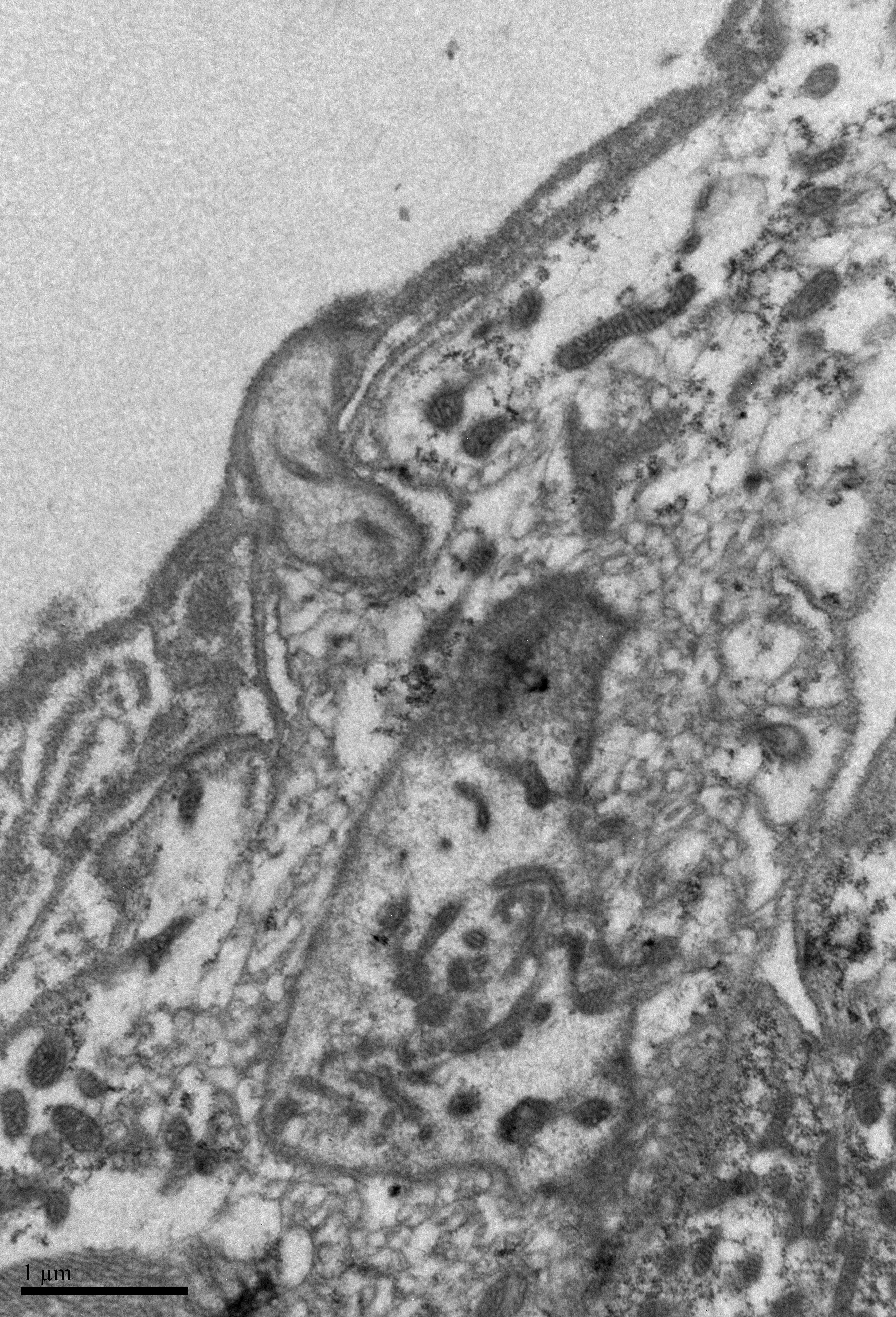

Supplement: Figure 1—source data 2. [file elife-46421-fig1-data2.zip › EM_no_driver_ctrl/003E-01A-bx2D2-007.jpg]

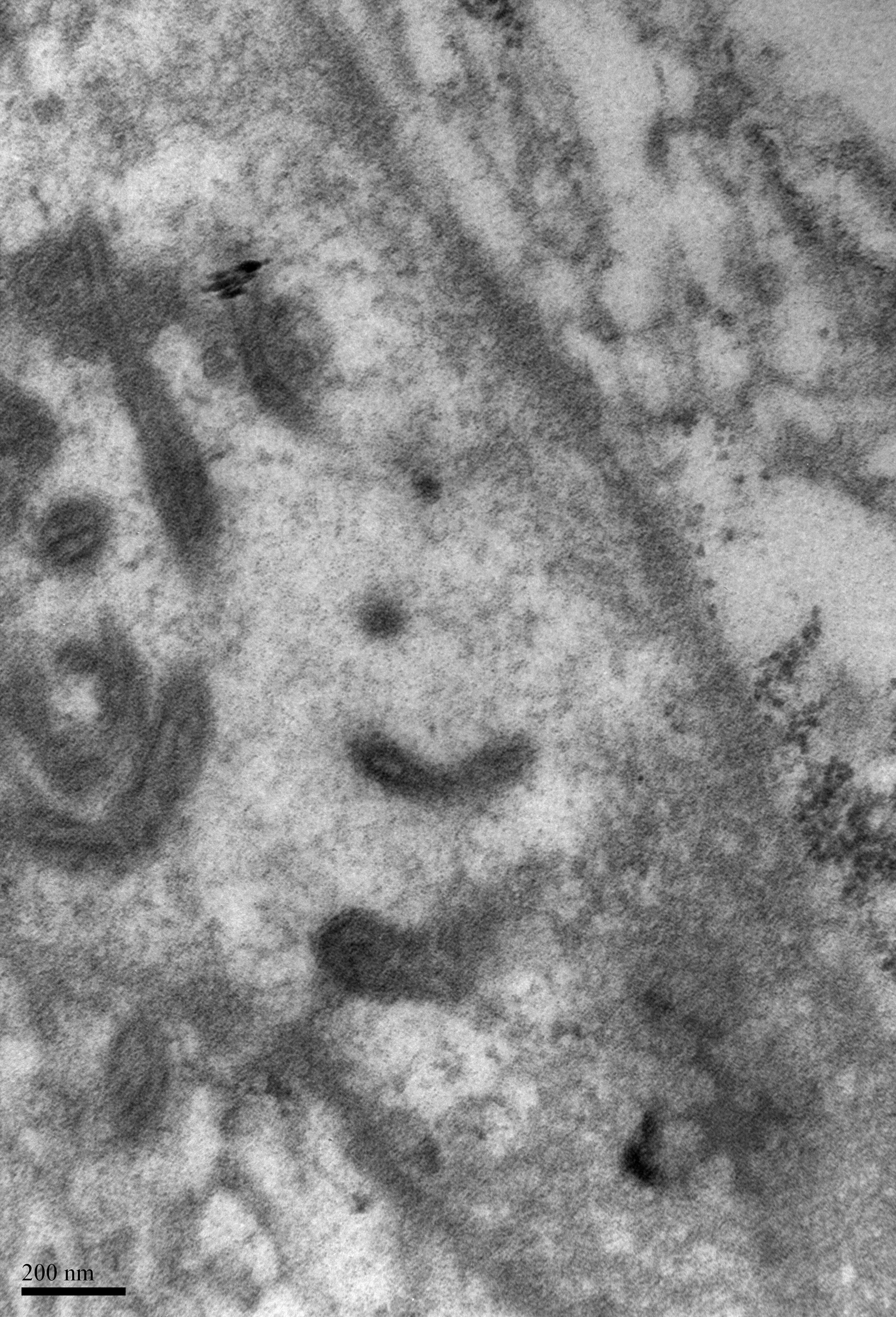

Supplement: Figure 1—source data 2. [file elife-46421-fig1-data2.zip › EM_no_driver_ctrl/003E-01A-bx2D2-008.jpg]

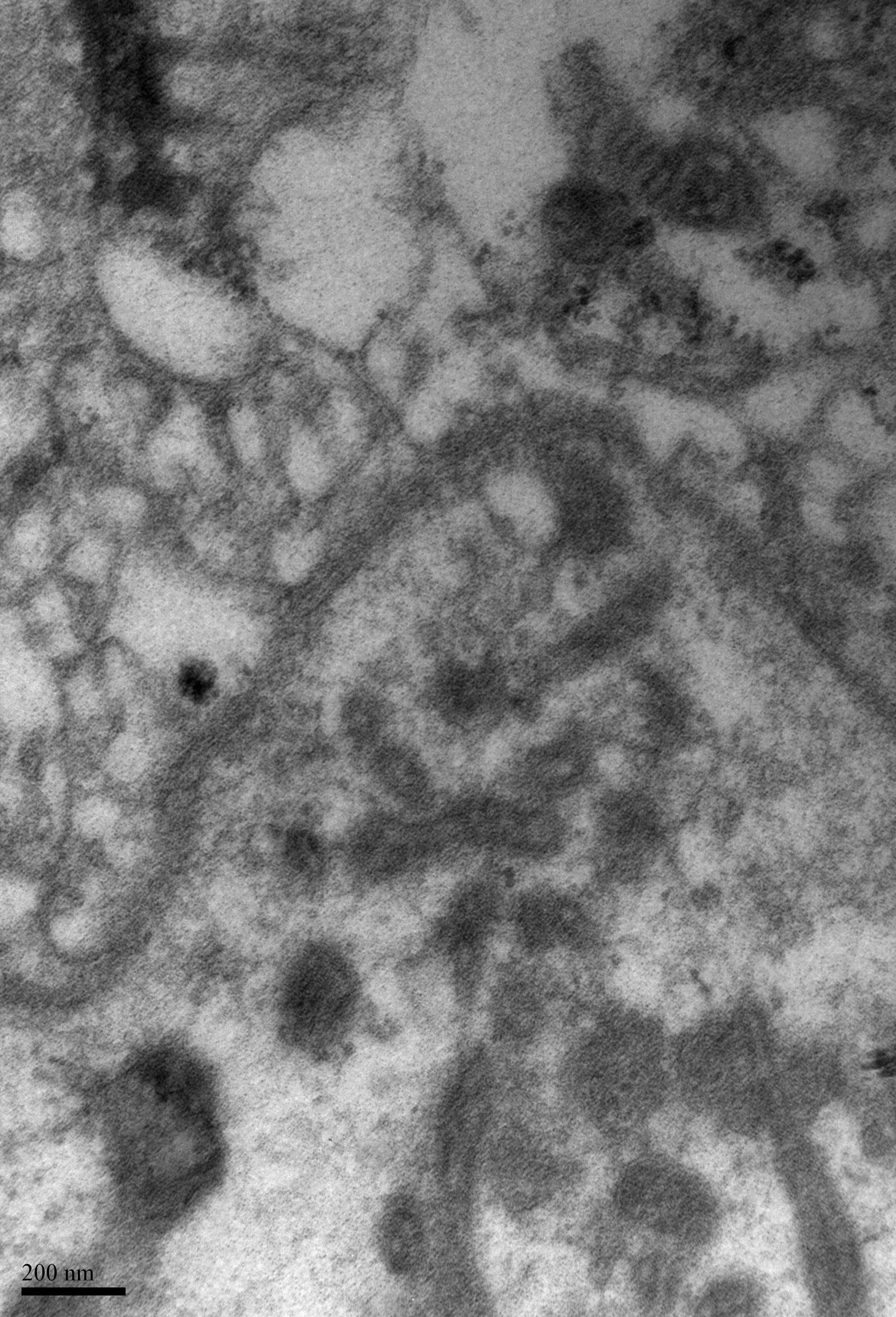

Supplement: Figure 1—source data 2. [file elife-46421-fig1-data2.zip › EM_no_driver_ctrl/003E-01A-bx2D2-009.jpg]

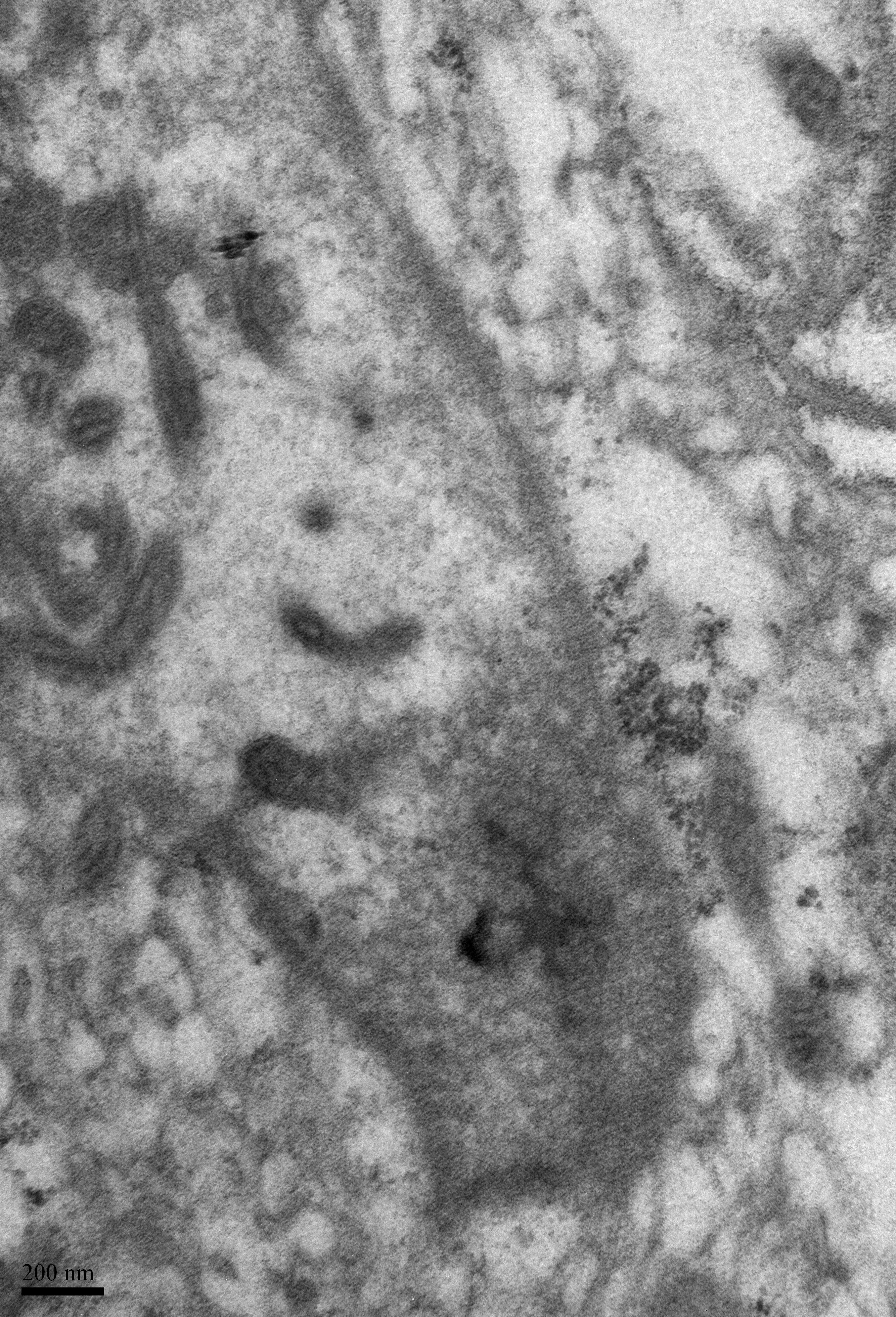

Supplement: Figure 1—source data 2. [file elife-46421-fig1-data2.zip › EM_no_driver_ctrl/003E-01A-bx2D2-010.jpg]

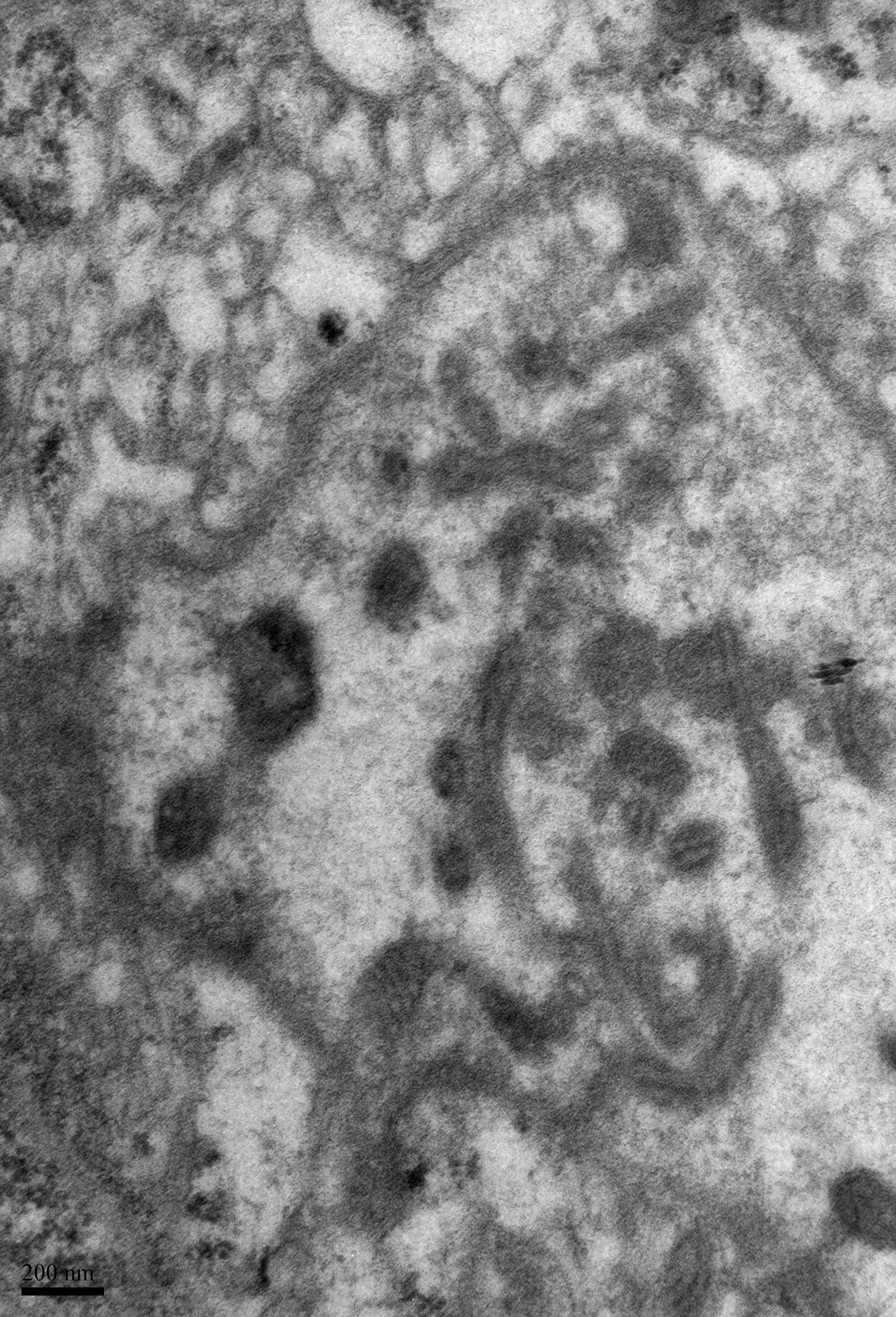

Supplement: Figure 1—source data 2. [file elife-46421-fig1-data2.zip › EM_no_driver_ctrl/003E-01A-bx2D2-011.jpg]

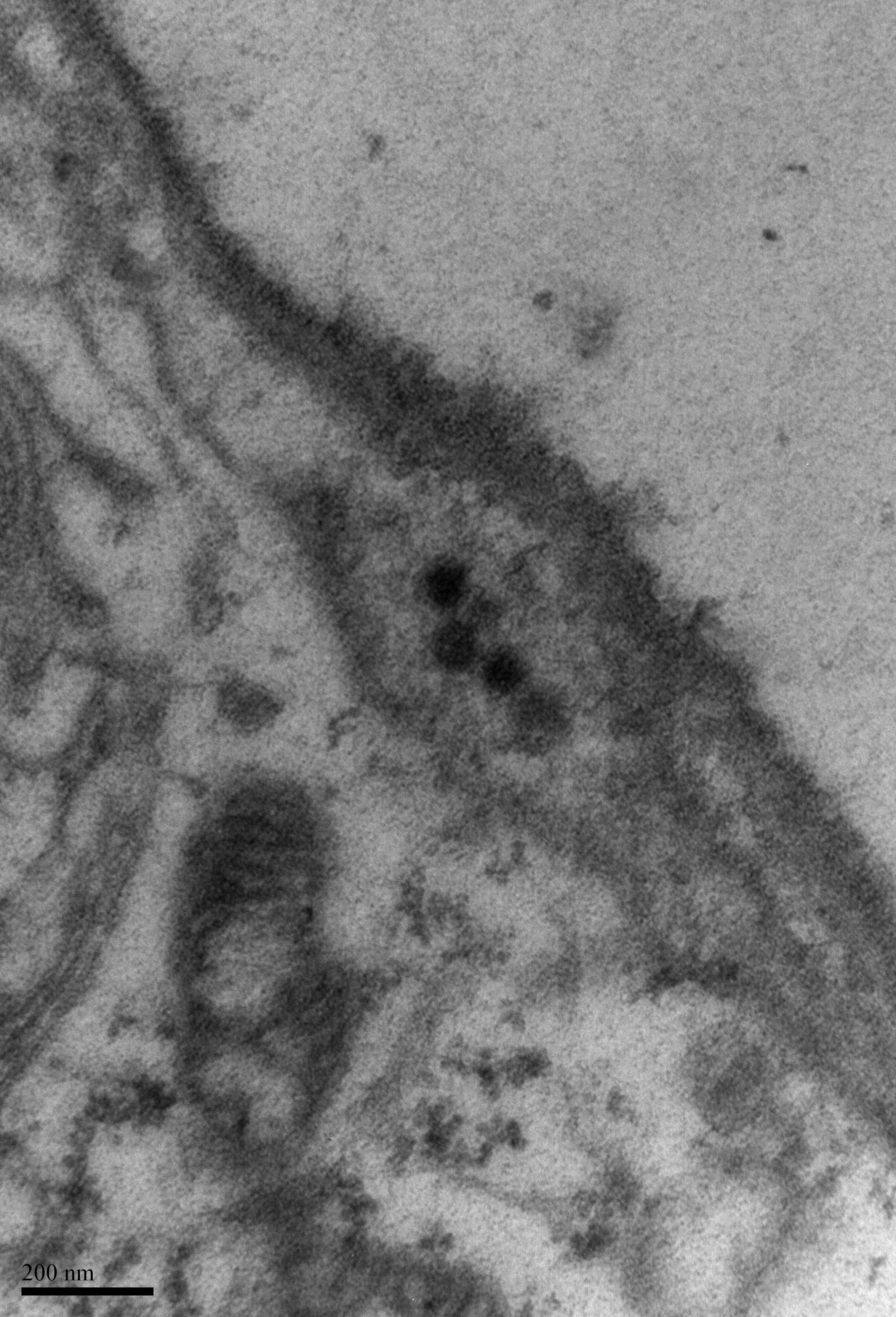

Supplement: Figure 1—source data 2. [file elife-46421-fig1-data2.zip › EM_no_driver_ctrl/003E-01A-bx2D2-012.jpg]

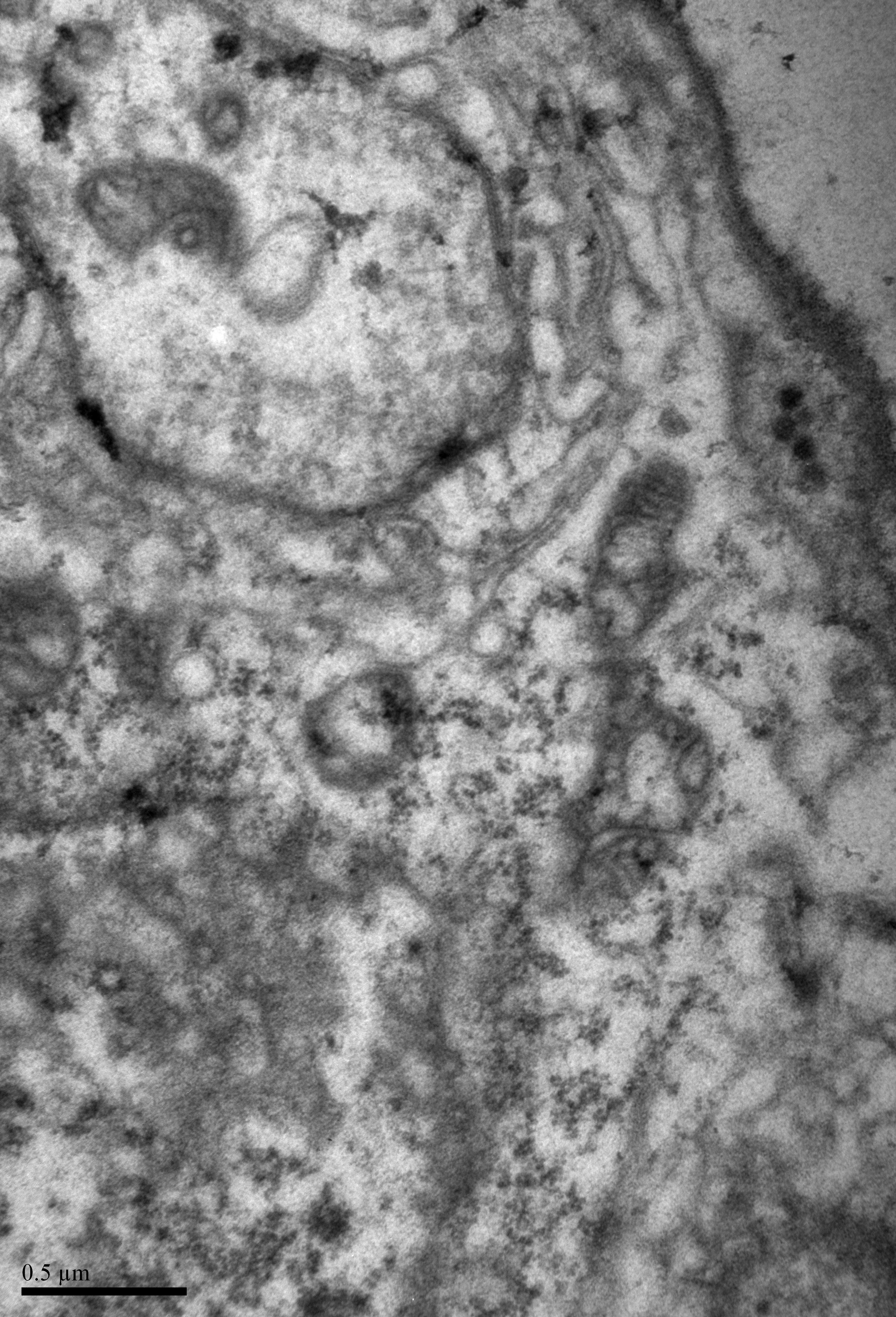

Supplement: Figure 1—source data 2. [file elife-46421-fig1-data2.zip › EM_no_driver_ctrl/003E-01A-bx2D2-013.jpg]

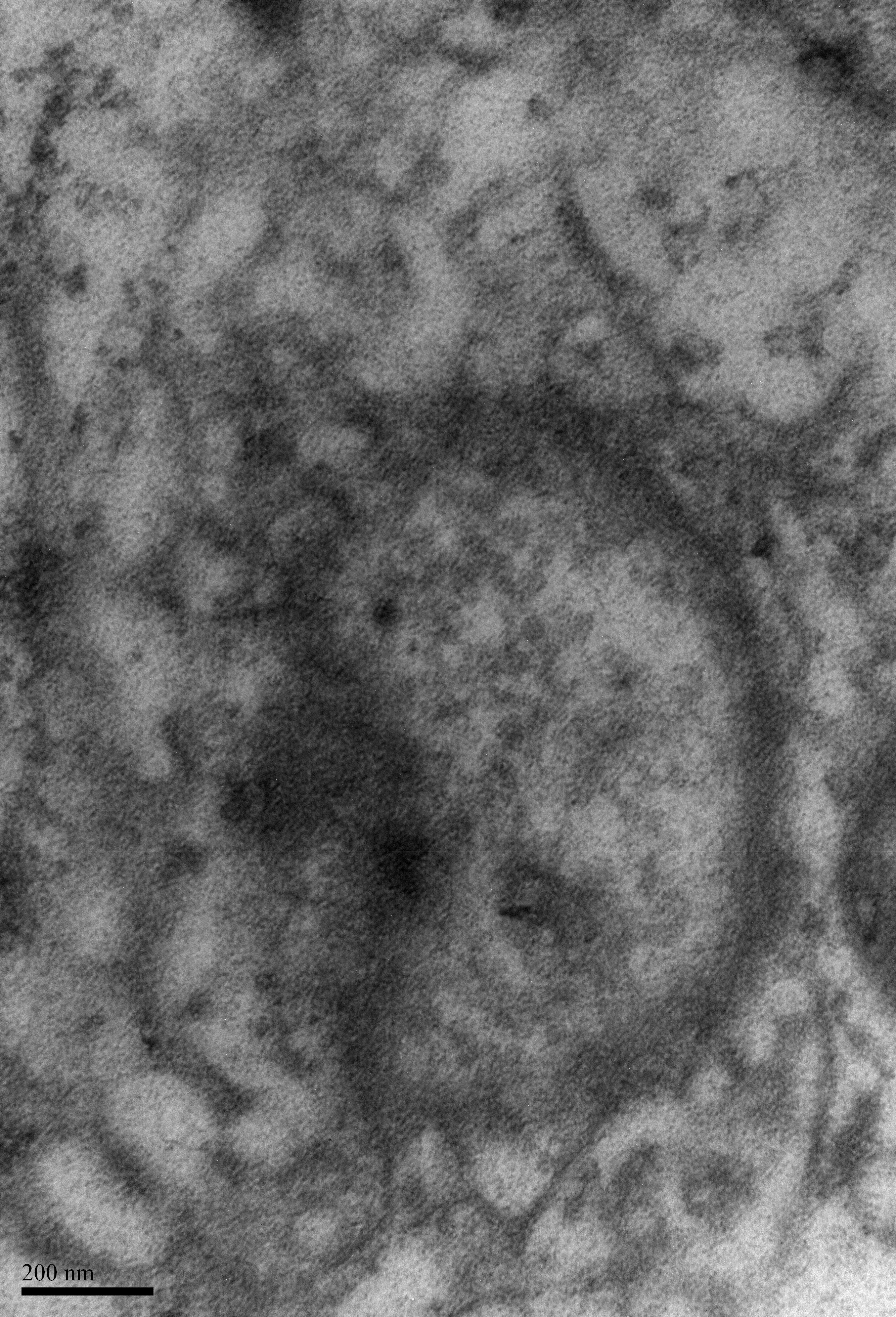

Supplement: Figure 1—source data 2. [file elife-46421-fig1-data2.zip › EM_no_driver_ctrl/003E-01A-bx2D2-014.jpg]

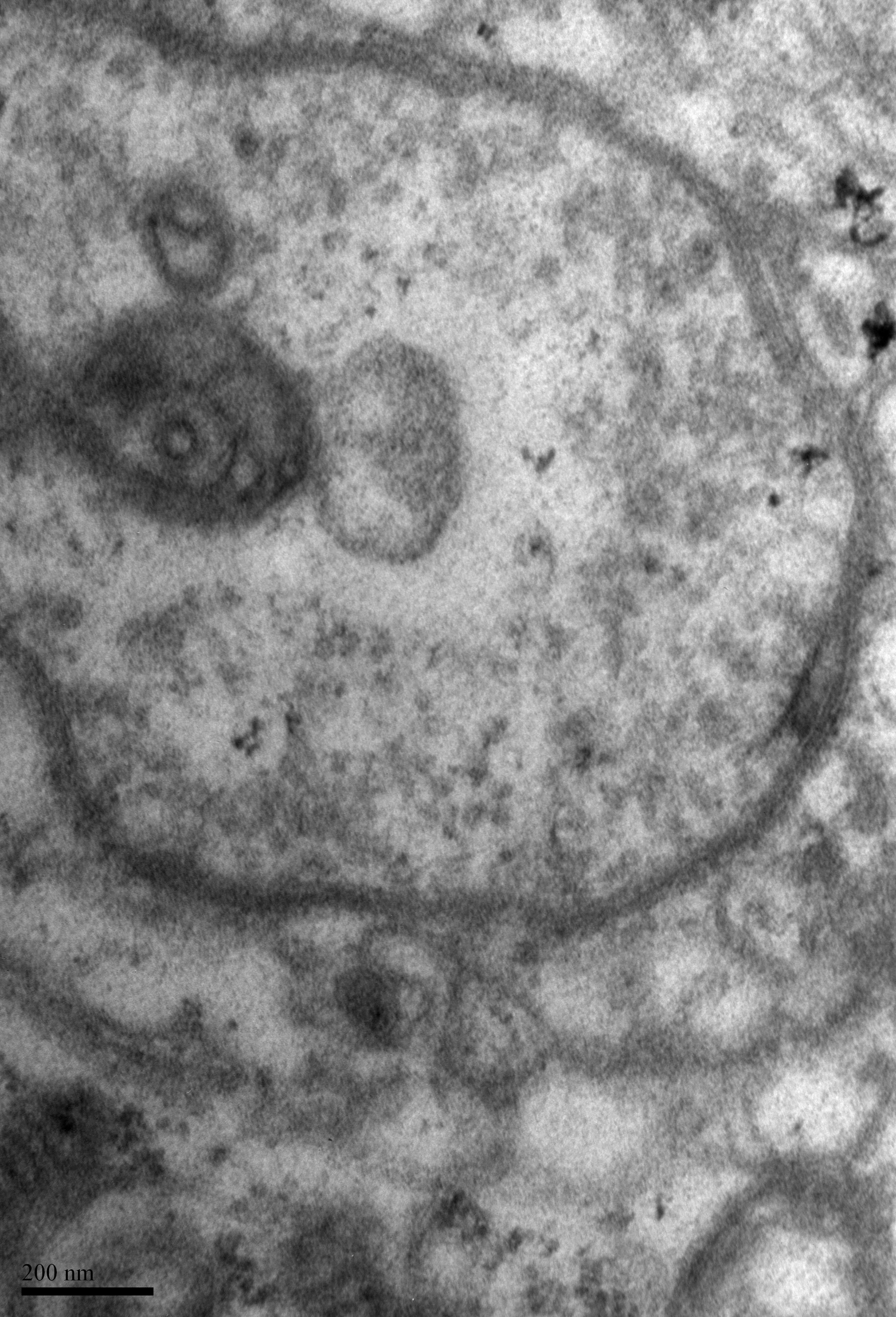

Supplement: Figure 1—source data 2. [file elife-46421-fig1-data2.zip › EM_no_driver_ctrl/003E-01A-bx2D2-015.jpg]

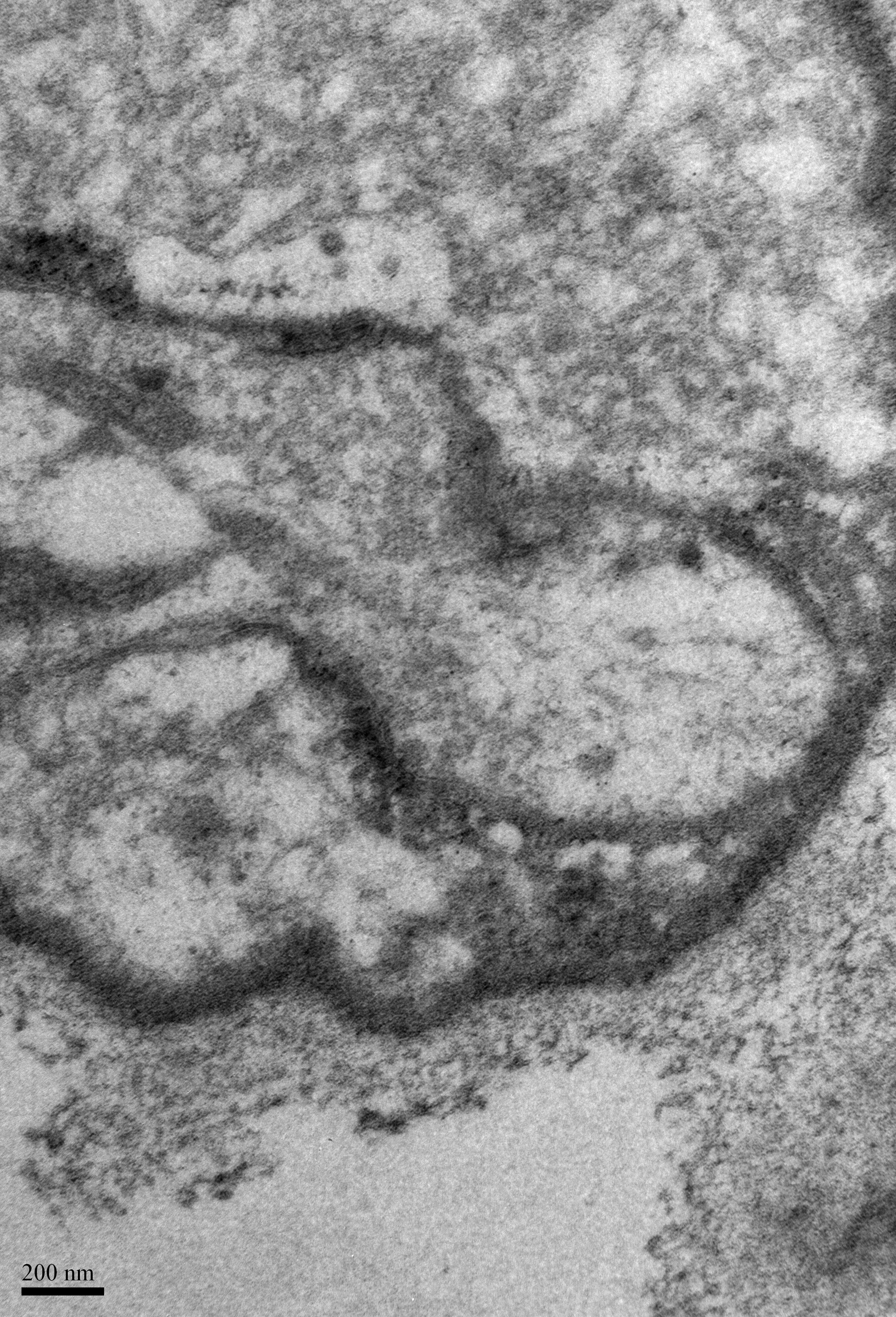

Supplement: Figure 3—source data 1. [file elife-46421-fig3-data1.zip › EM_NPRRdTK/003D-01B-bx2A5-001.jpg]

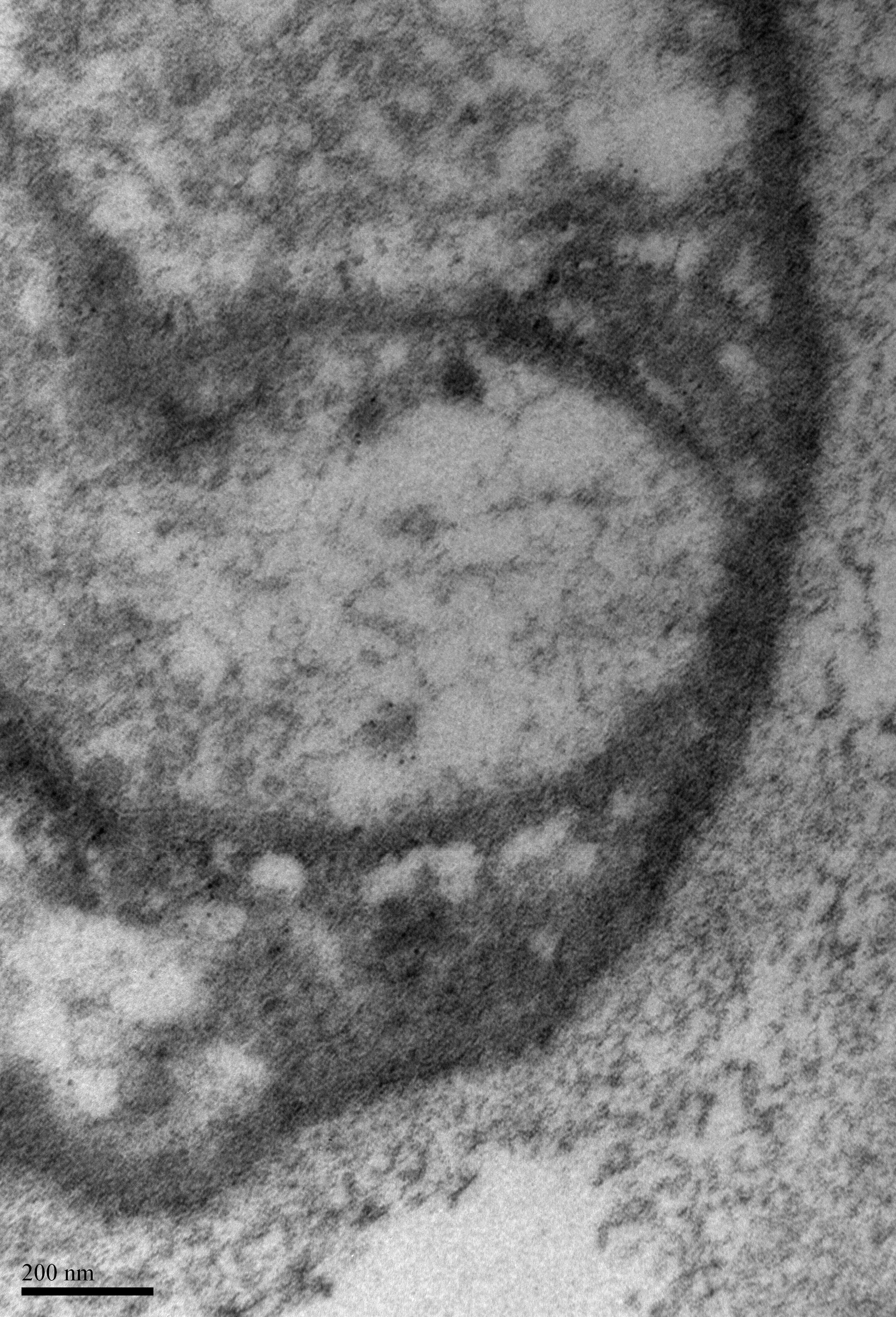

Supplement: Figure 3—source data 1. [file elife-46421-fig3-data1.zip › EM_NPRRdTK/003D-01B-bx2A5-002.jpg]

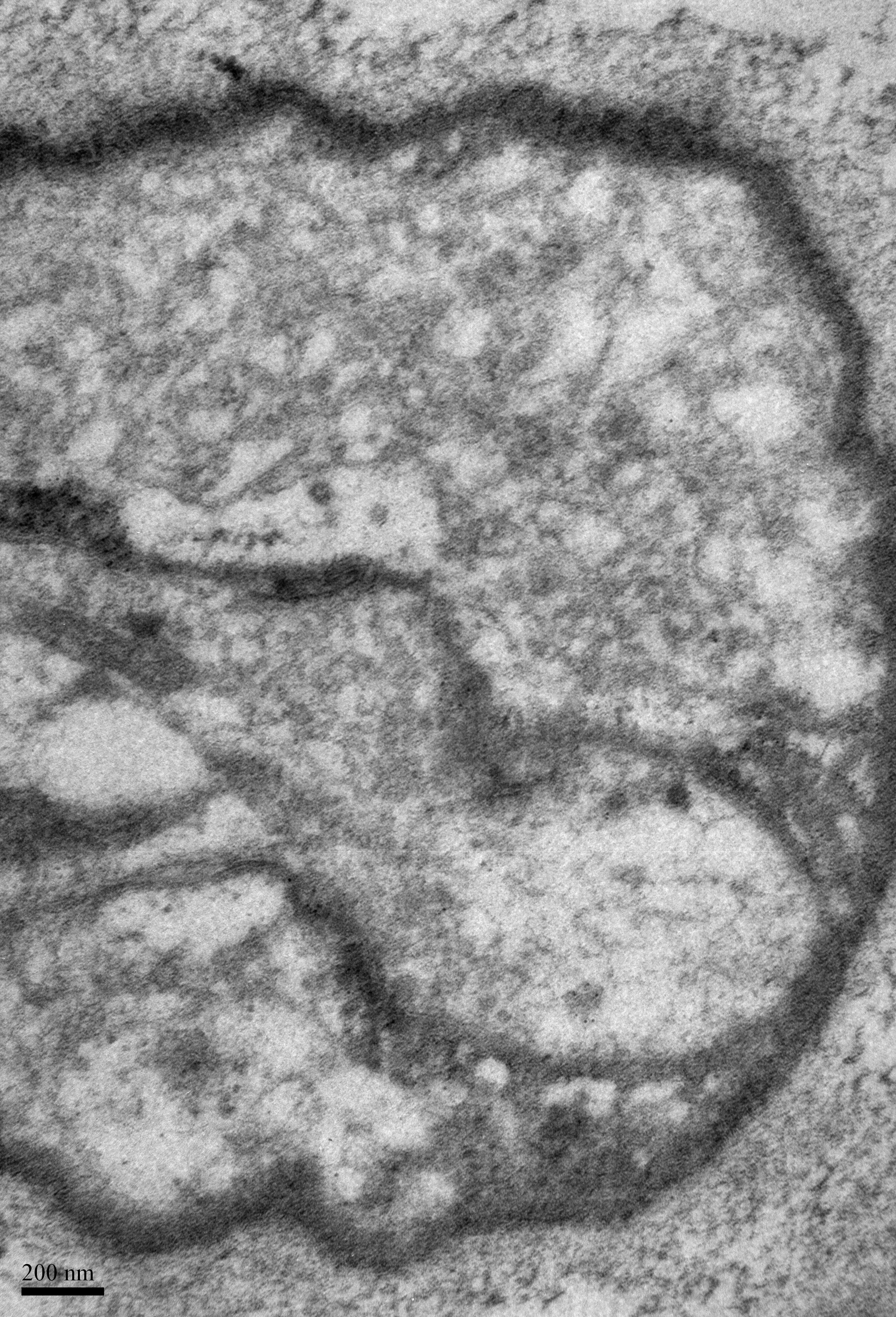

Supplement: Figure 3—source data 1. [file elife-46421-fig3-data1.zip › EM_NPRRdTK/003D-01B-bx2A5-003.jpg]

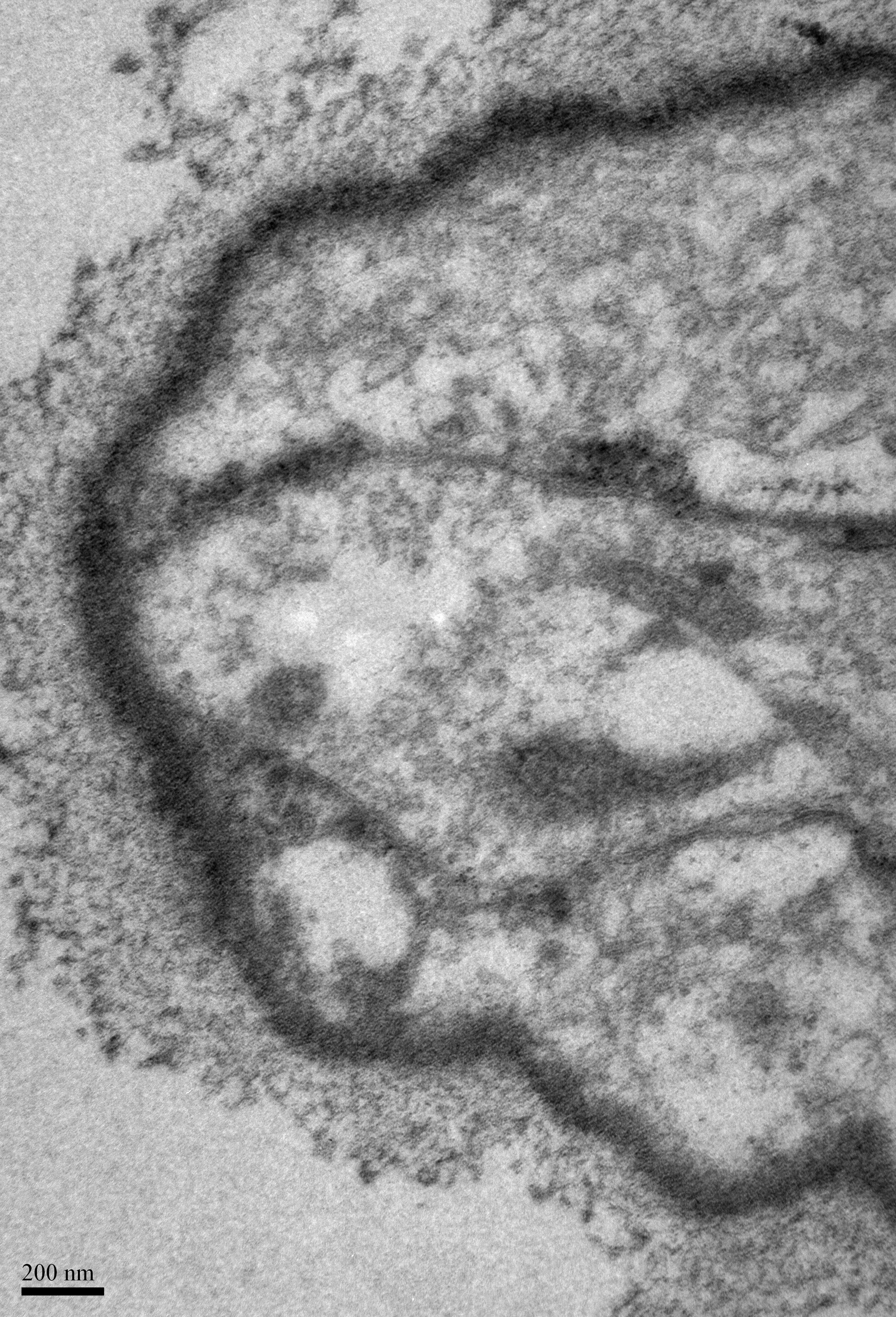

Supplement: Figure 3—source data 1. [file elife-46421-fig3-data1.zip › EM_NPRRdTK/003D-01B-bx2A5-004.jpg]

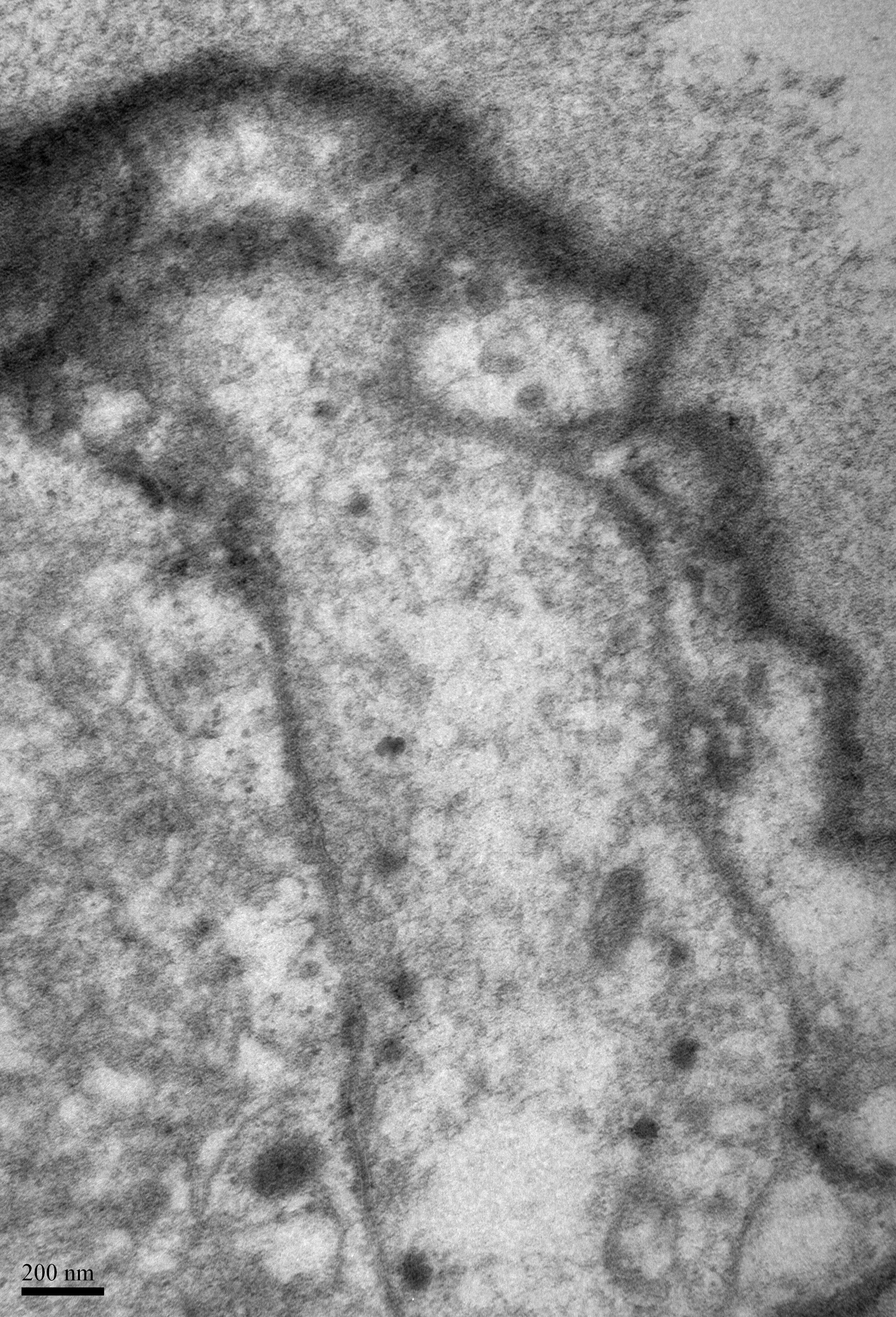

Supplement: Figure 3—source data 1. [file elife-46421-fig3-data1.zip › EM_NPRRdTK/003D-01B-bx2A5-005.jpg]

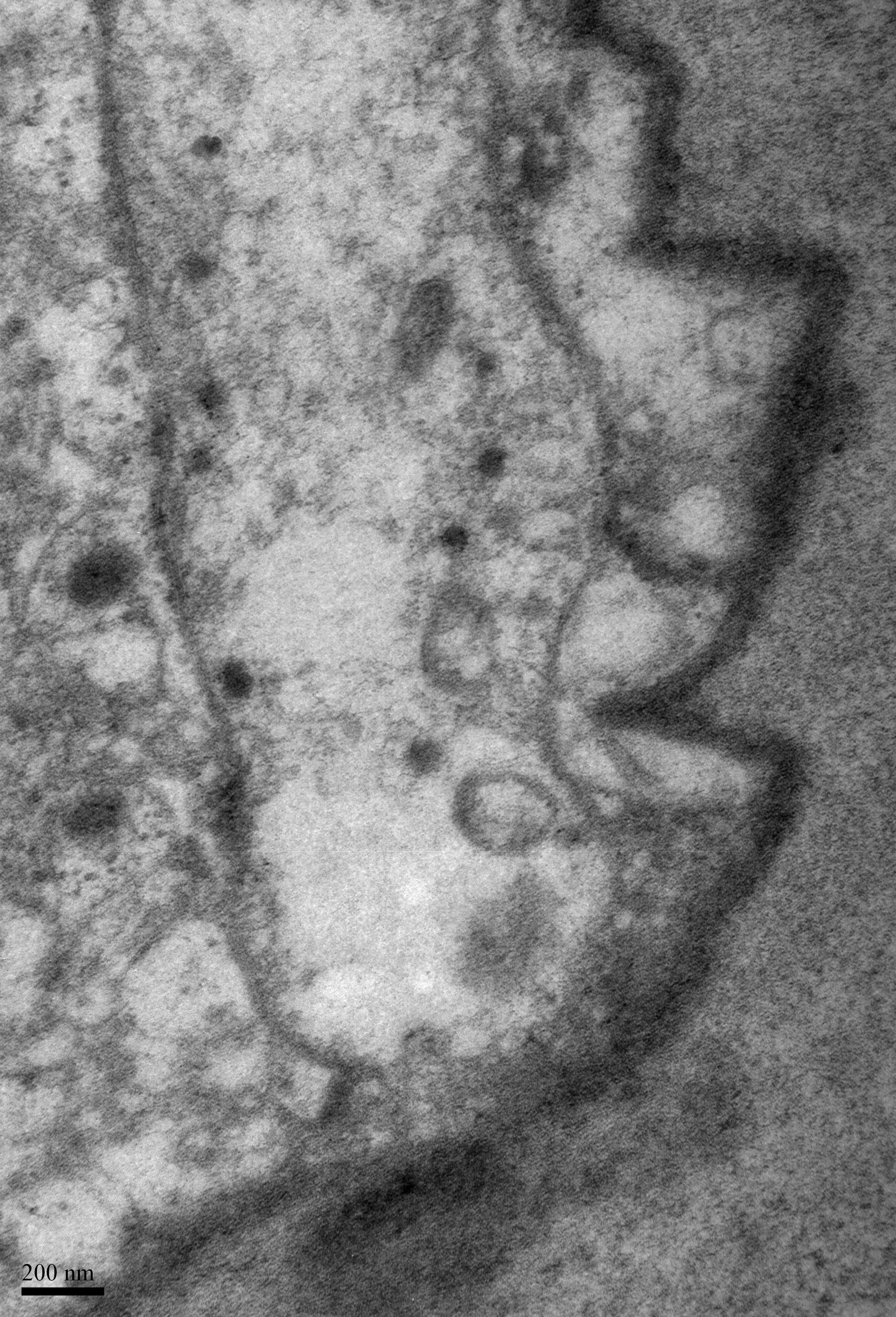

Supplement: Figure 3—source data 1. [file elife-46421-fig3-data1.zip › EM_NPRRdTK/003D-01B-bx2A5-006.jpg]

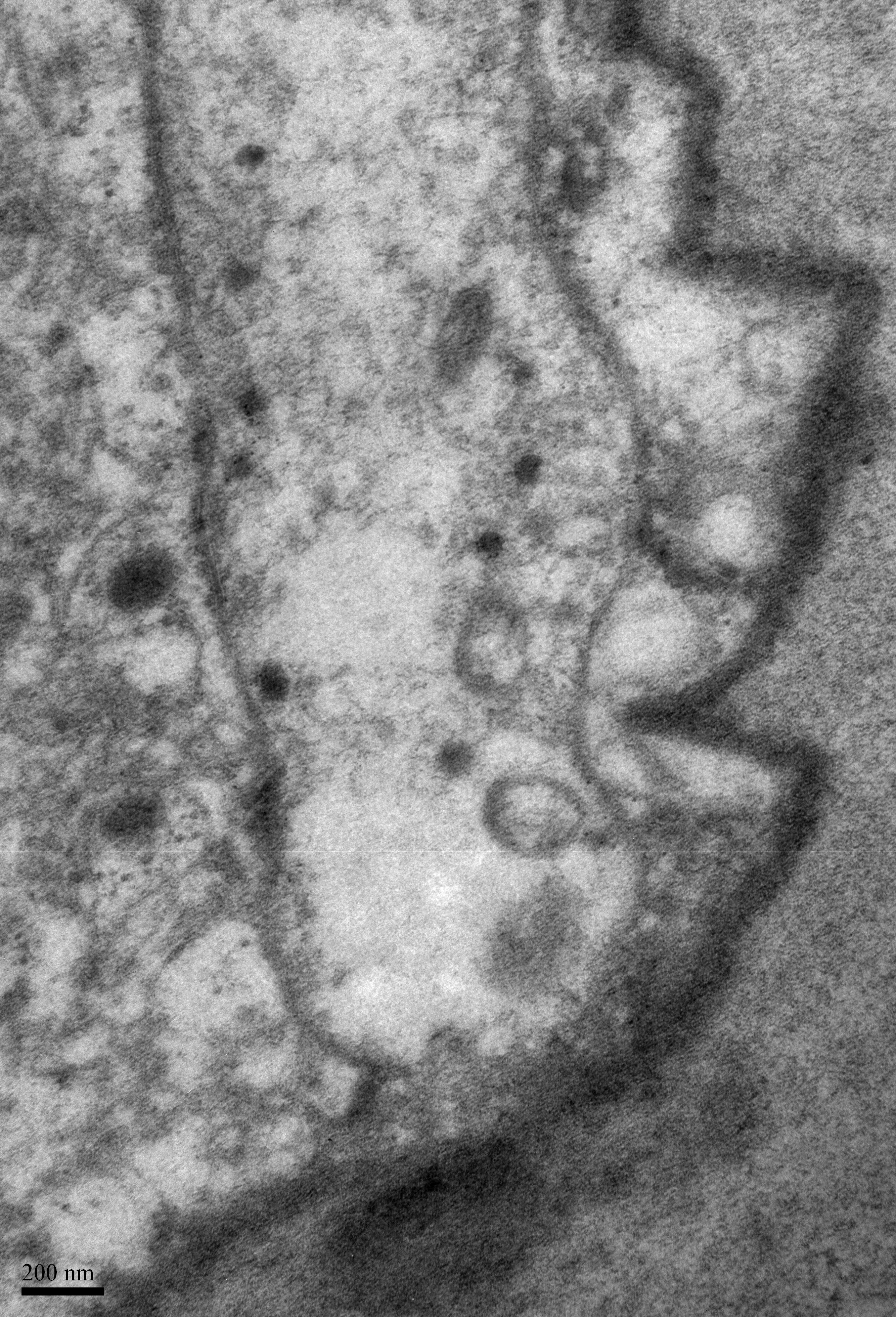

Supplement: Figure 3—source data 1. [file elife-46421-fig3-data1.zip › EM_NPRRdTK/003D-01B-bx2A5-007.jpg]

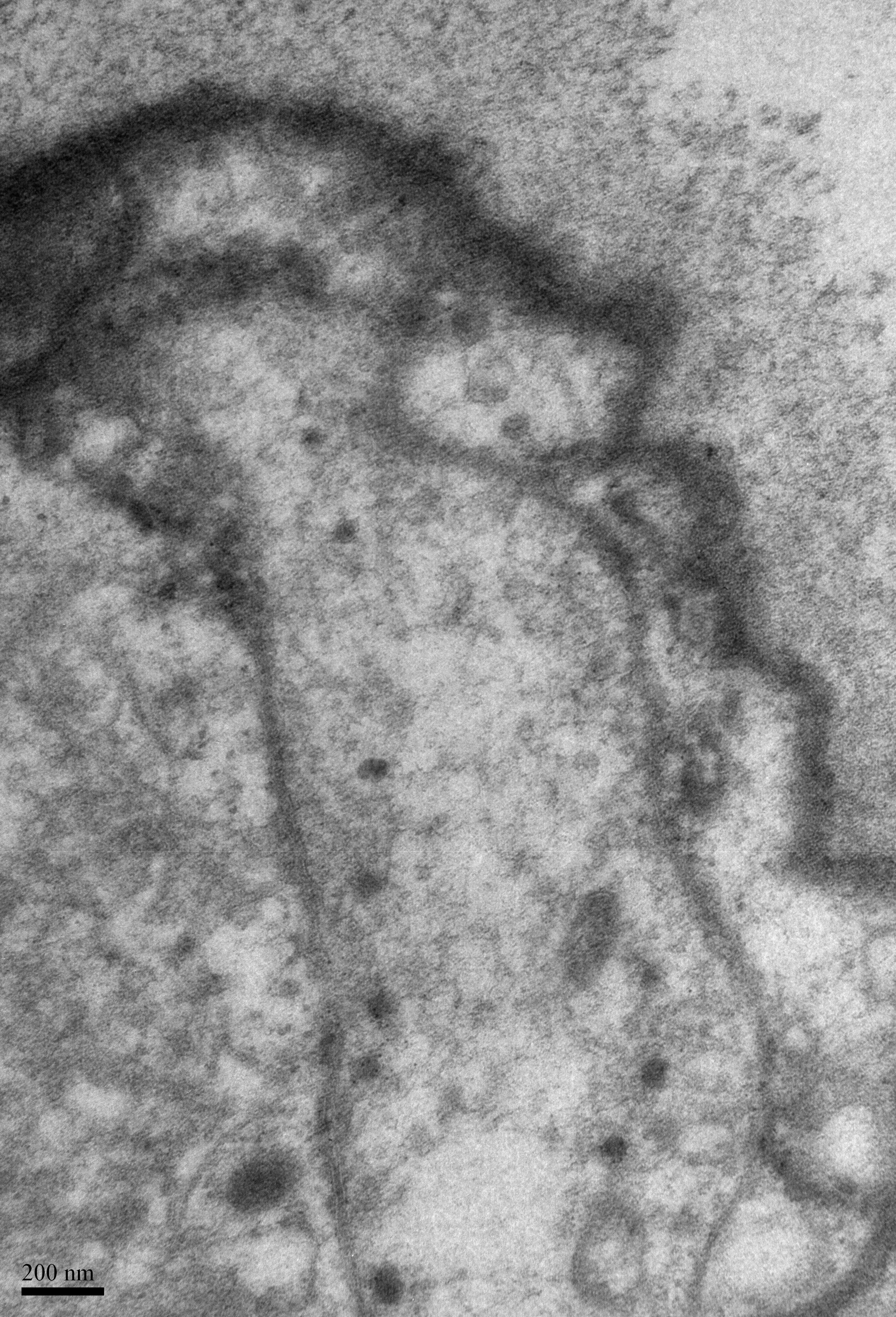

Supplement: Figure 3—source data 1. [file elife-46421-fig3-data1.zip › EM_NPRRdTK/003D-01B-bx2A5-008.jpg]

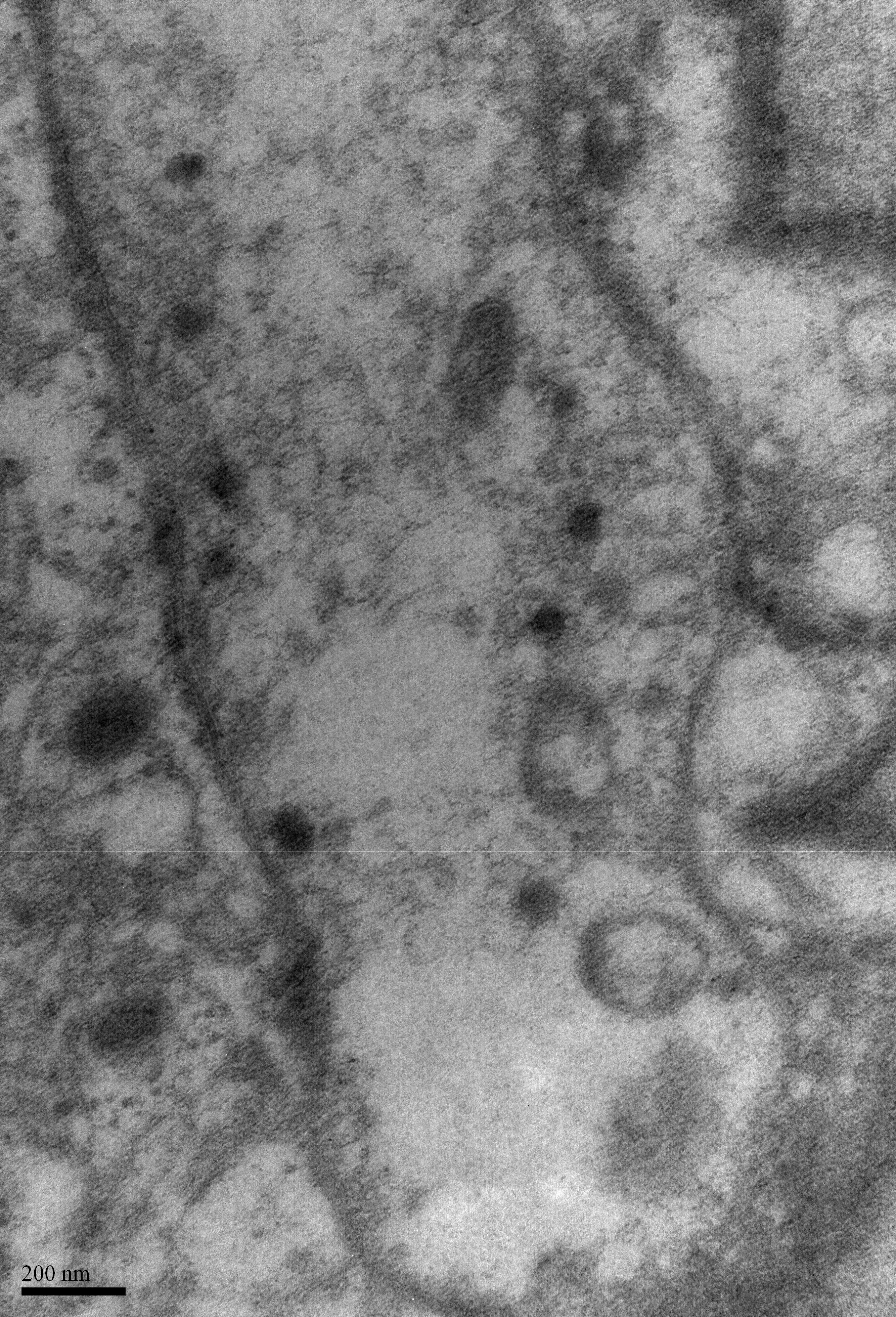

Supplement: Figure 3—source data 1. [file elife-46421-fig3-data1.zip › EM_NPRRdTK/003D-01B-bx2A5-009.jpg]

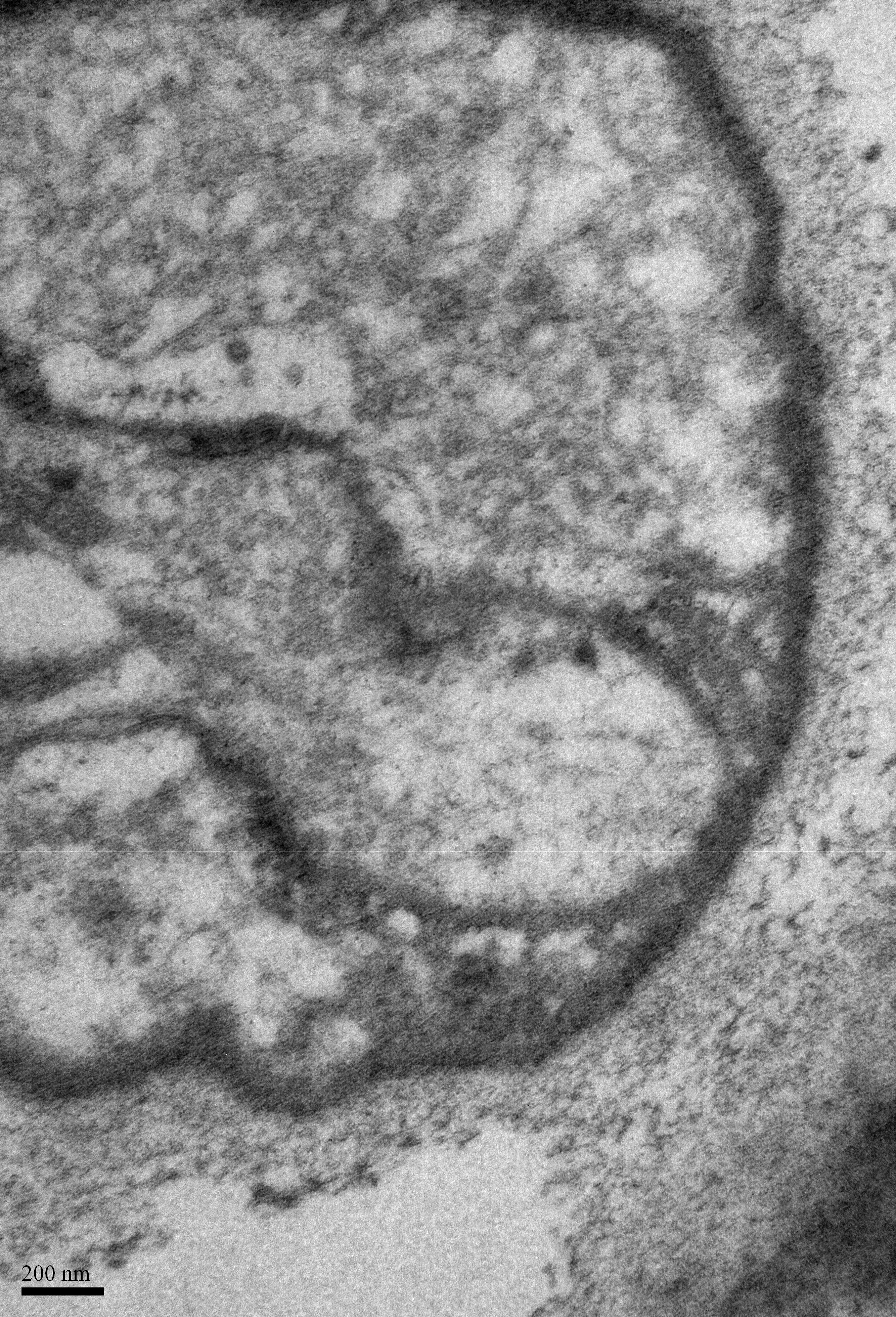

Supplement: Figure 3—source data 1. [file elife-46421-fig3-data1.zip › EM_NPRRdTK/003D-01B-bx2A5-010.jpg]

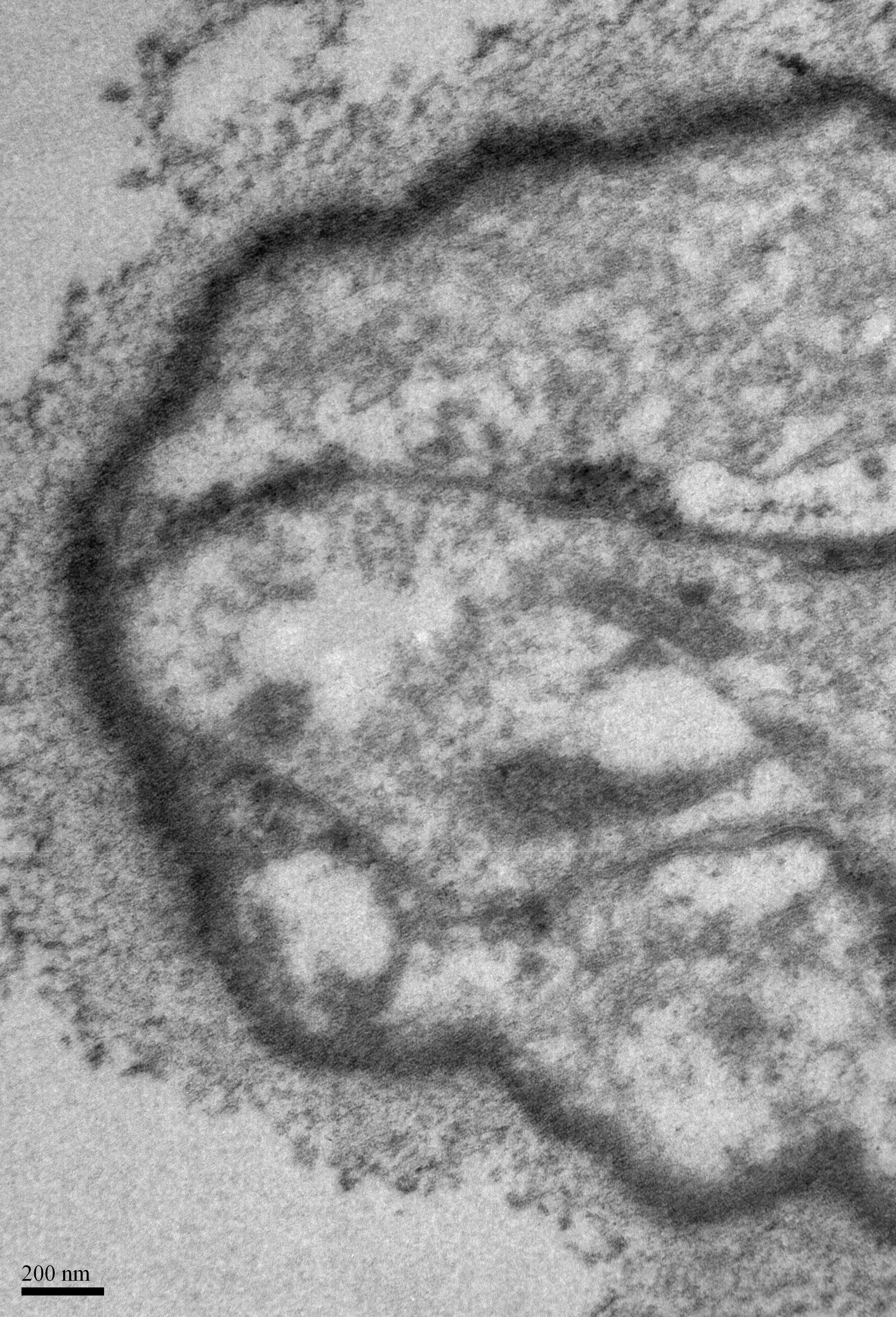

Supplement: Figure 3—source data 1. [file elife-46421-fig3-data1.zip › EM_NPRRdTK/003D-01B-bx2A5-011.jpg]

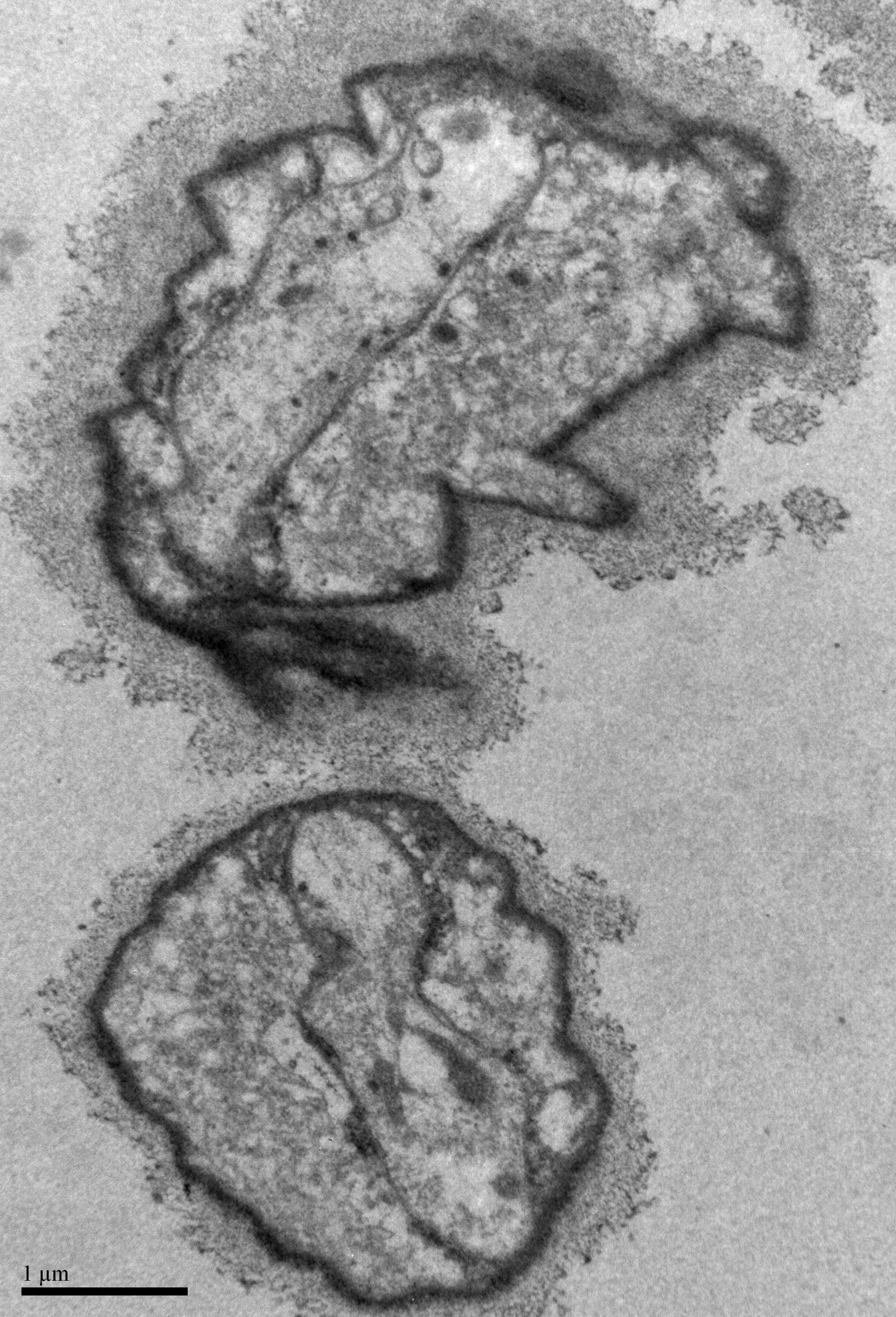

Supplement: Figure 3—source data 1. [file elife-46421-fig3-data1.zip › EM_NPRRdTK/003D-01B-bx2A5-012.jpg]

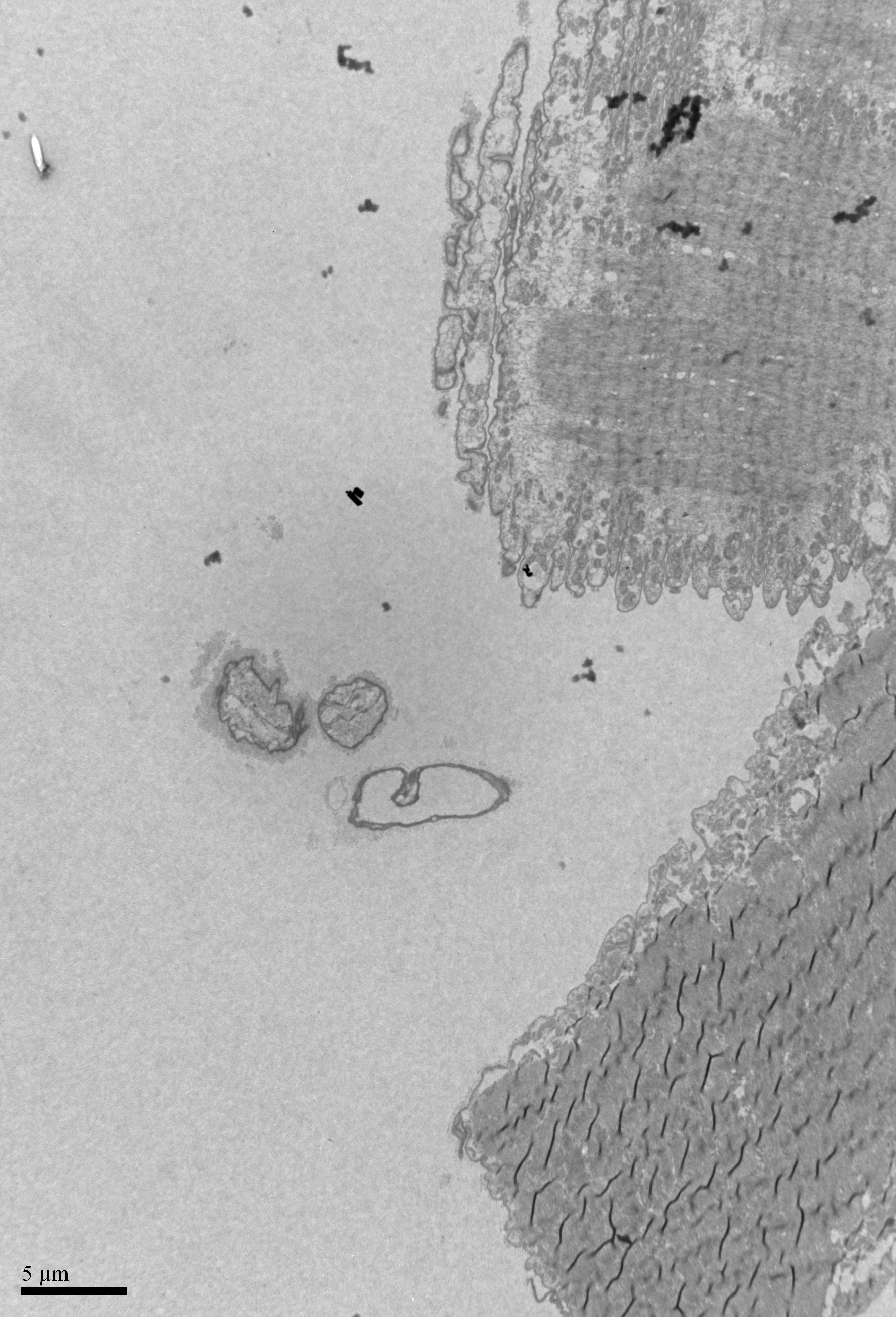

Supplement: Figure 3—source data 1. [file elife-46421-fig3-data1.zip › EM_NPRRdTK/003D-01B-bx2A5-013.jpg]

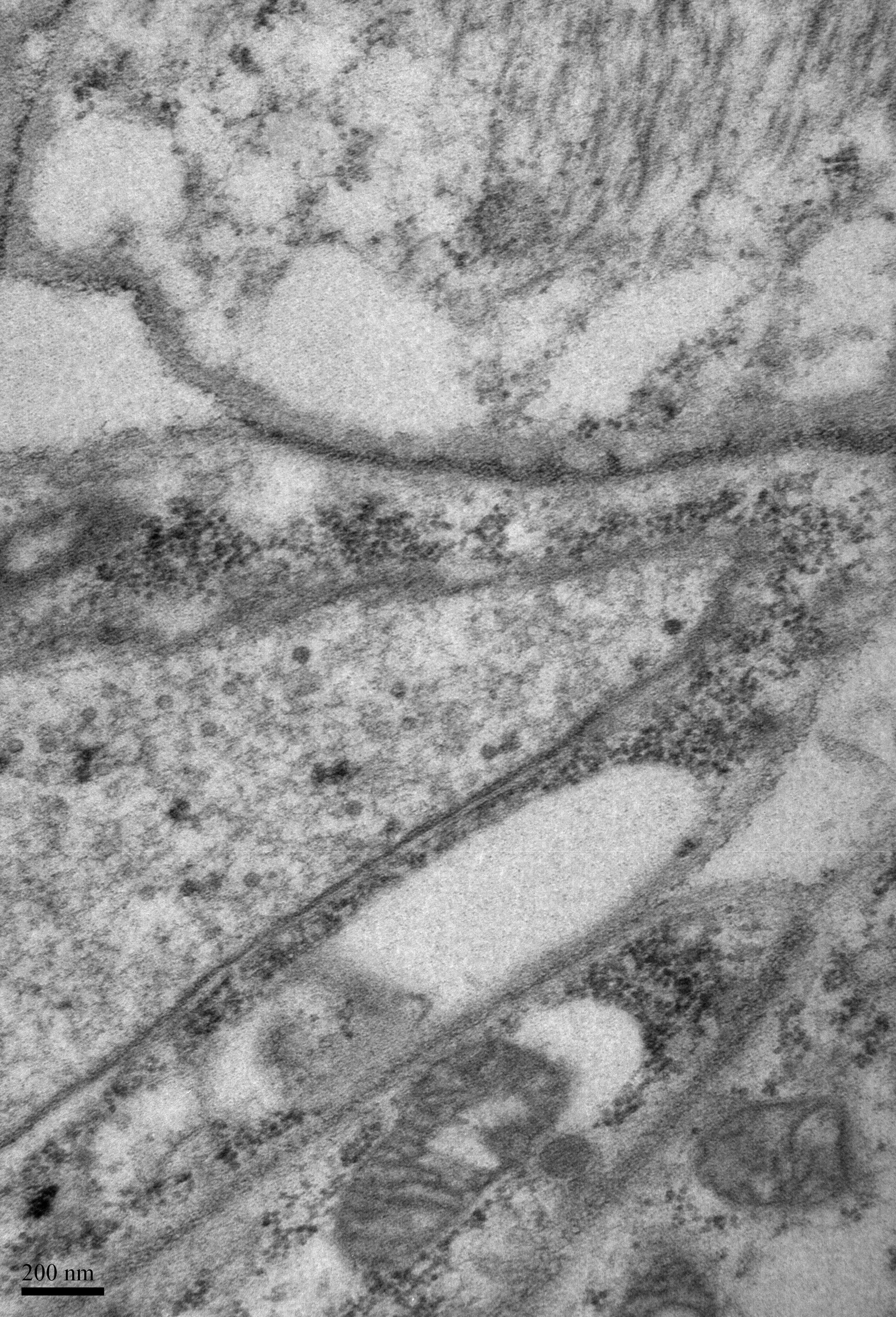

Supplement: Figure 3—source data 1. [file elife-46421-fig3-data1.zip › EM_NPRRdTK/003D-01B-bx2A5-014.jpg]

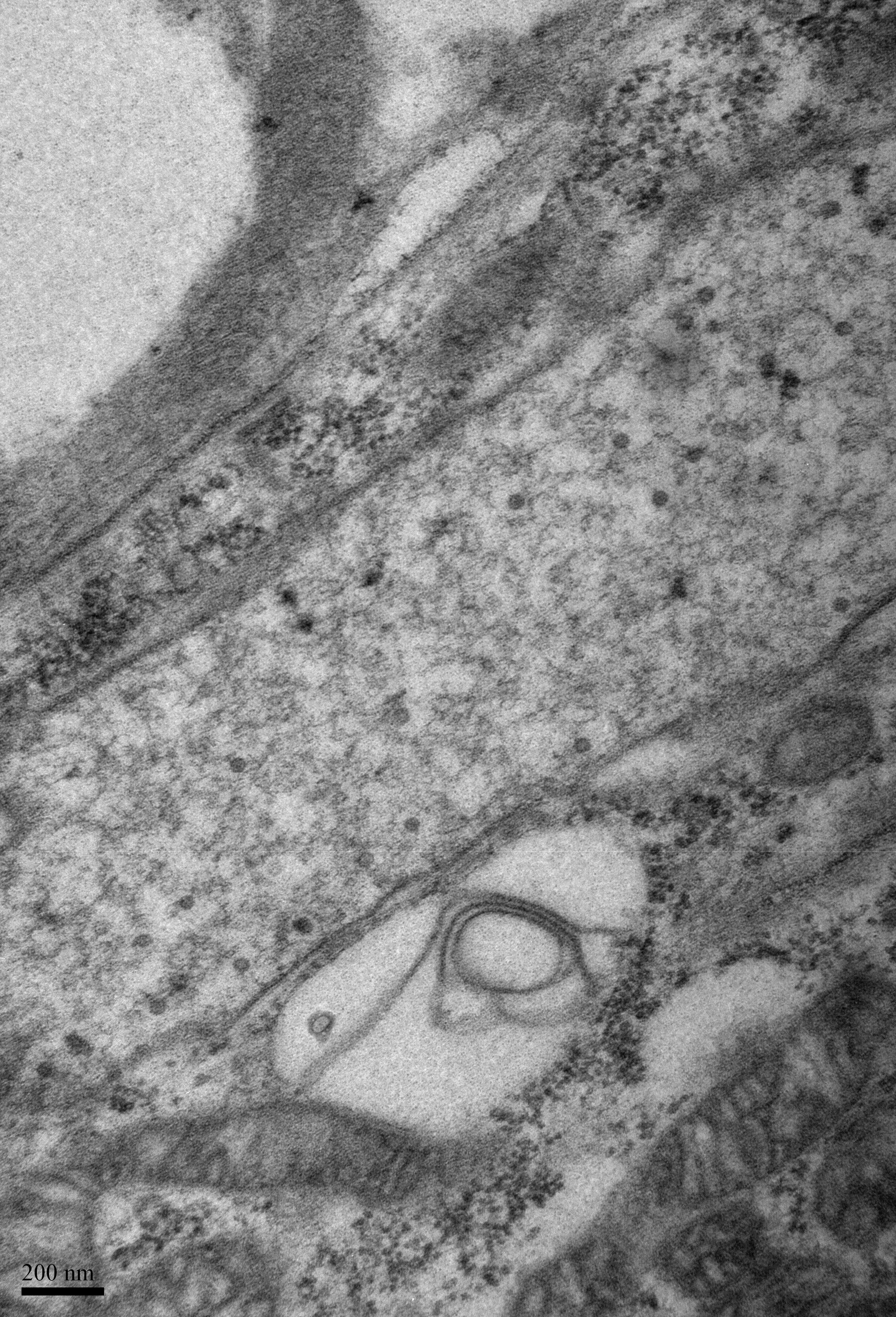

Supplement: Figure 3—source data 1. [file elife-46421-fig3-data1.zip › EM_NPRRdTK/003D-01B-bx2A5-015.jpg]

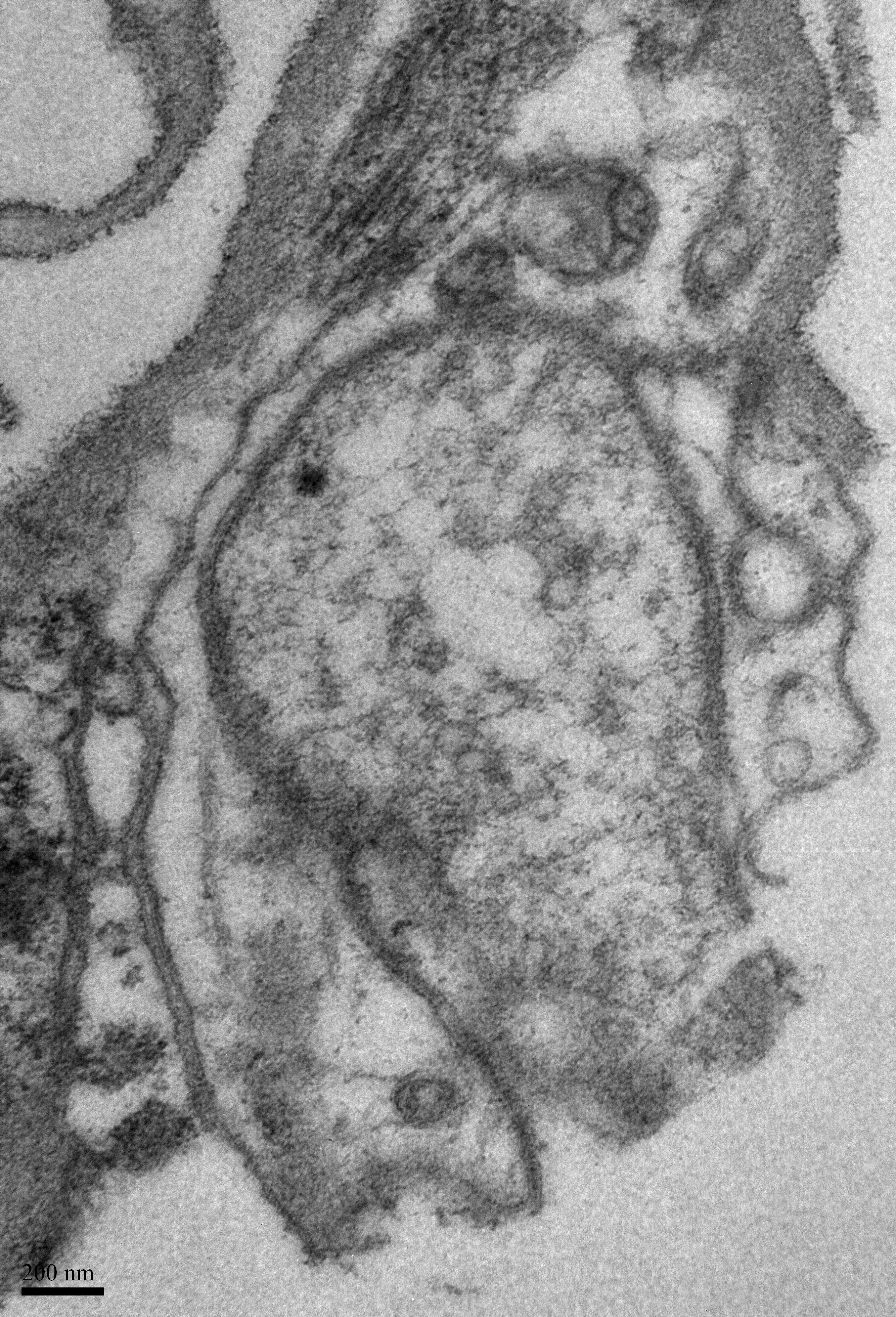

Supplement: Figure 3—source data 1. [file elife-46421-fig3-data1.zip › EM_NPRRdTK/003D-01B-bx2A5-016.jpg]

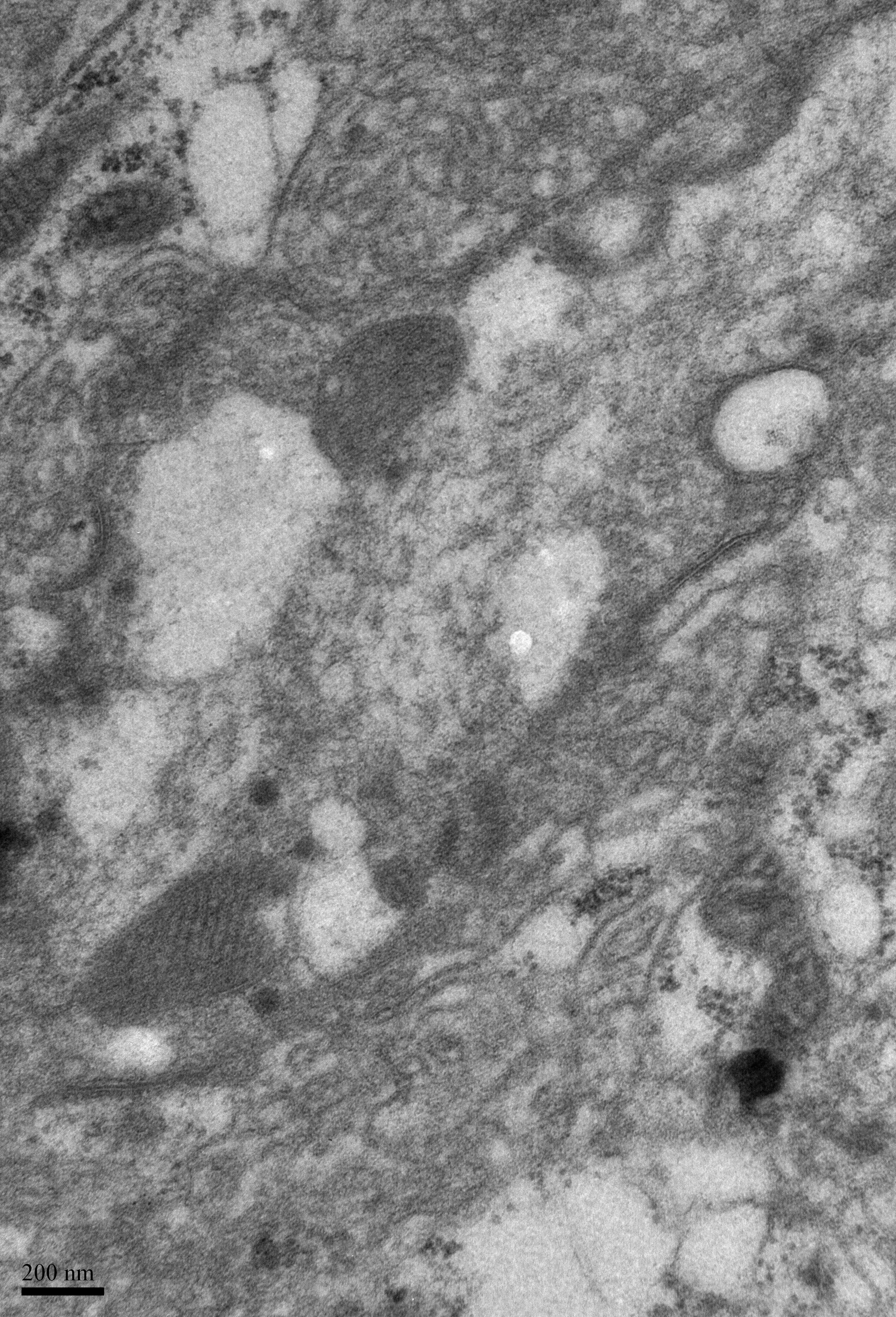

Supplement: Figure 3—source data 1. [file elife-46421-fig3-data1.zip › EM_NPRRdTK/003D-01B-bx2A5-017.jpg]

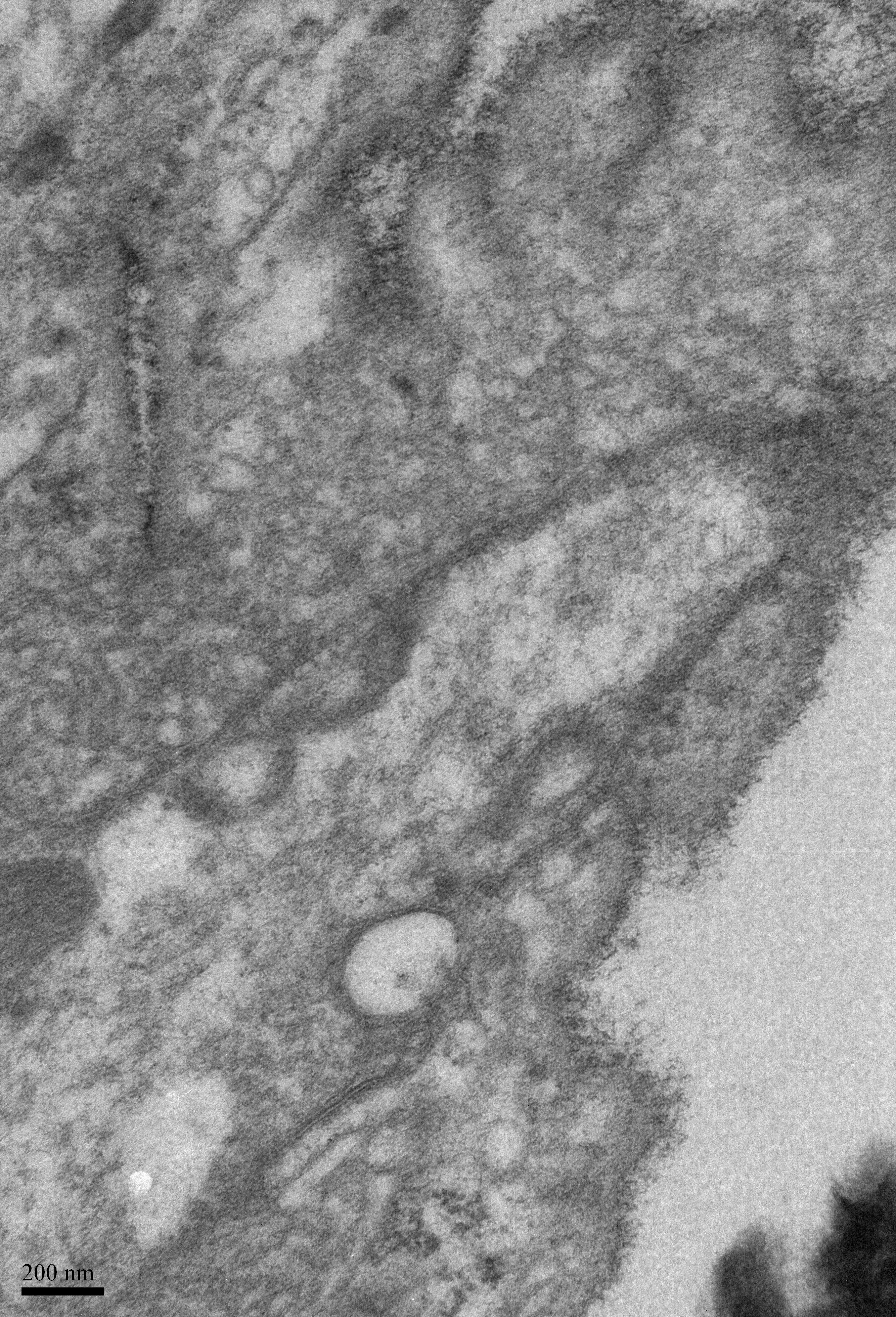

Supplement: Figure 3—source data 1. [file elife-46421-fig3-data1.zip › EM_NPRRdTK/003D-01B-bx2A5-018.jpg]

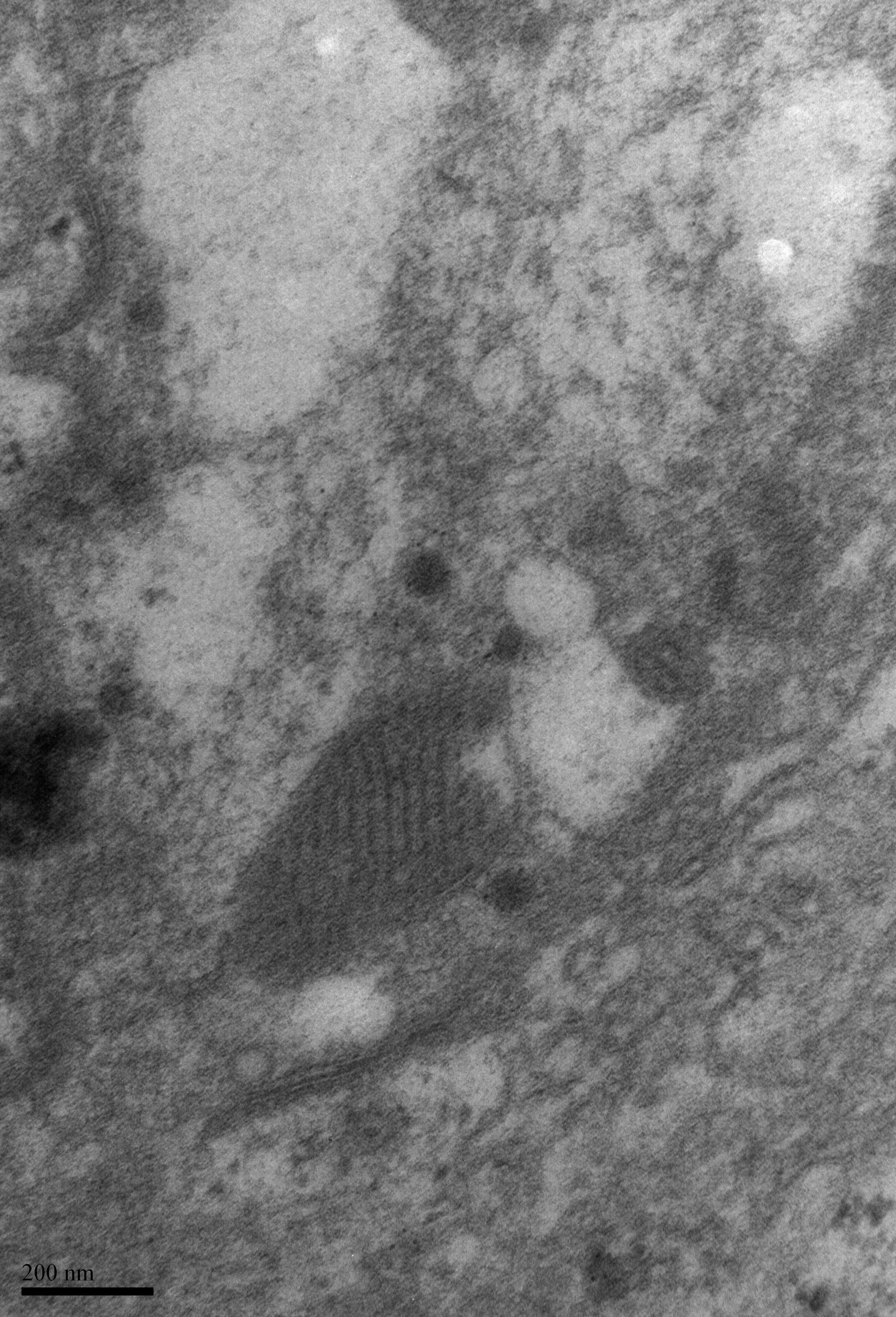

Supplement: Figure 3—source data 1. [file elife-46421-fig3-data1.zip › EM_NPRRdTK/003D-01B-bx2A5-019.jpg]

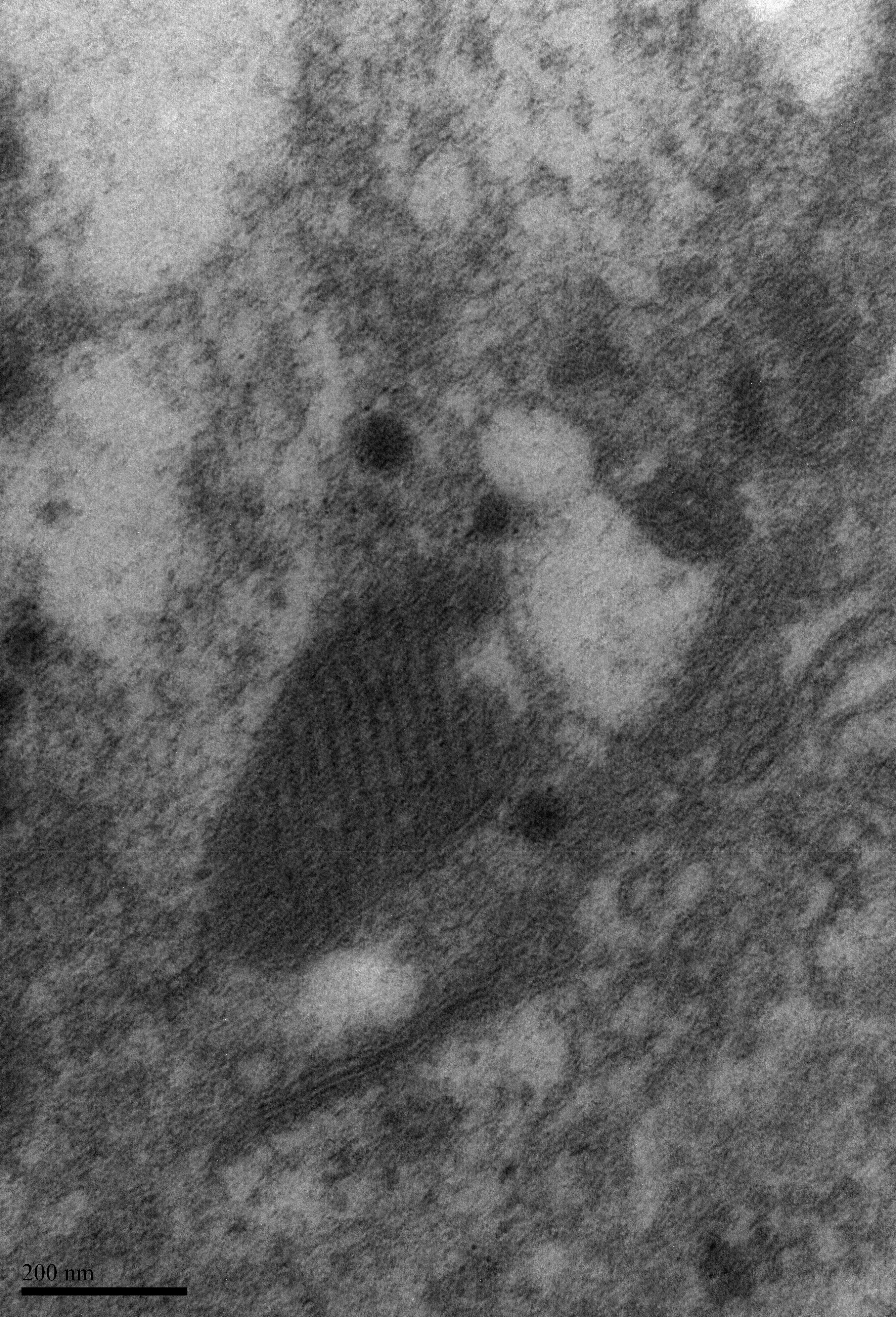

Supplement: Figure 3—source data 1. [file elife-46421-fig3-data1.zip › EM_NPRRdTK/003D-01B-bx2A5-020.jpg]

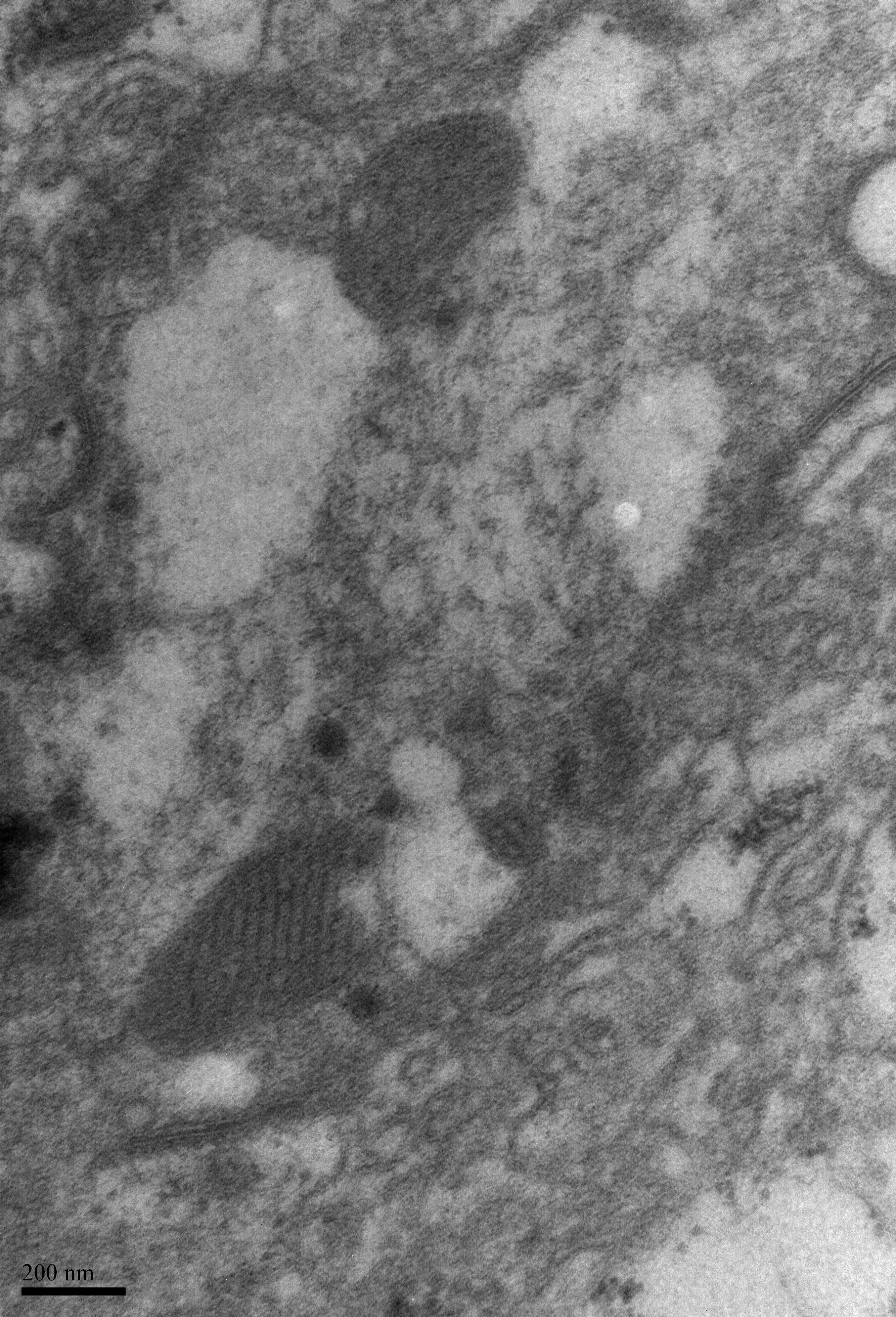

Supplement: Figure 3—source data 1. [file elife-46421-fig3-data1.zip › EM_NPRRdTK/003D-01B-bx2A5-021.jpg]

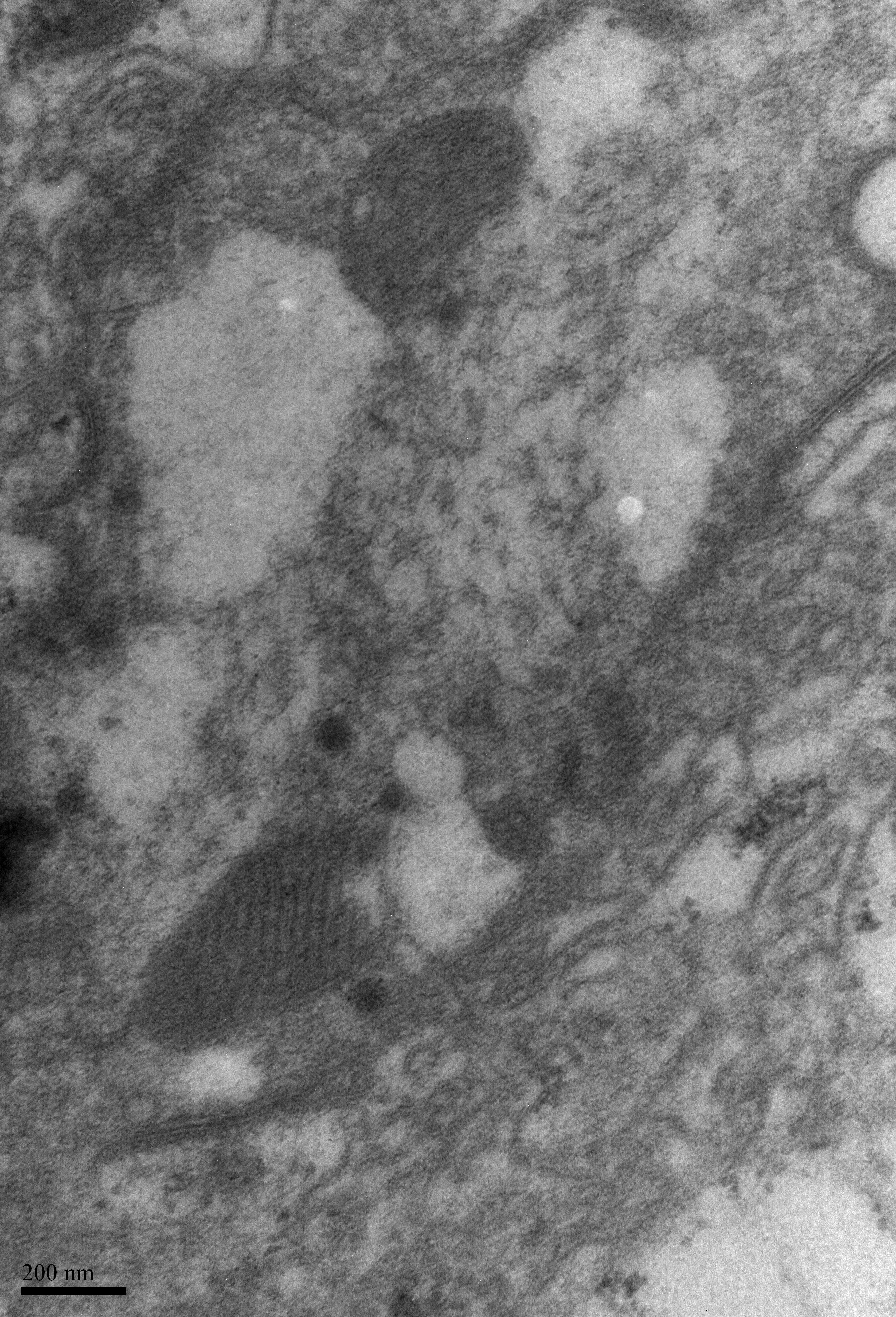

Supplement: Figure 3—source data 1. [file elife-46421-fig3-data1.zip › EM_NPRRdTK/003D-01B-bx2A5-022.jpg]

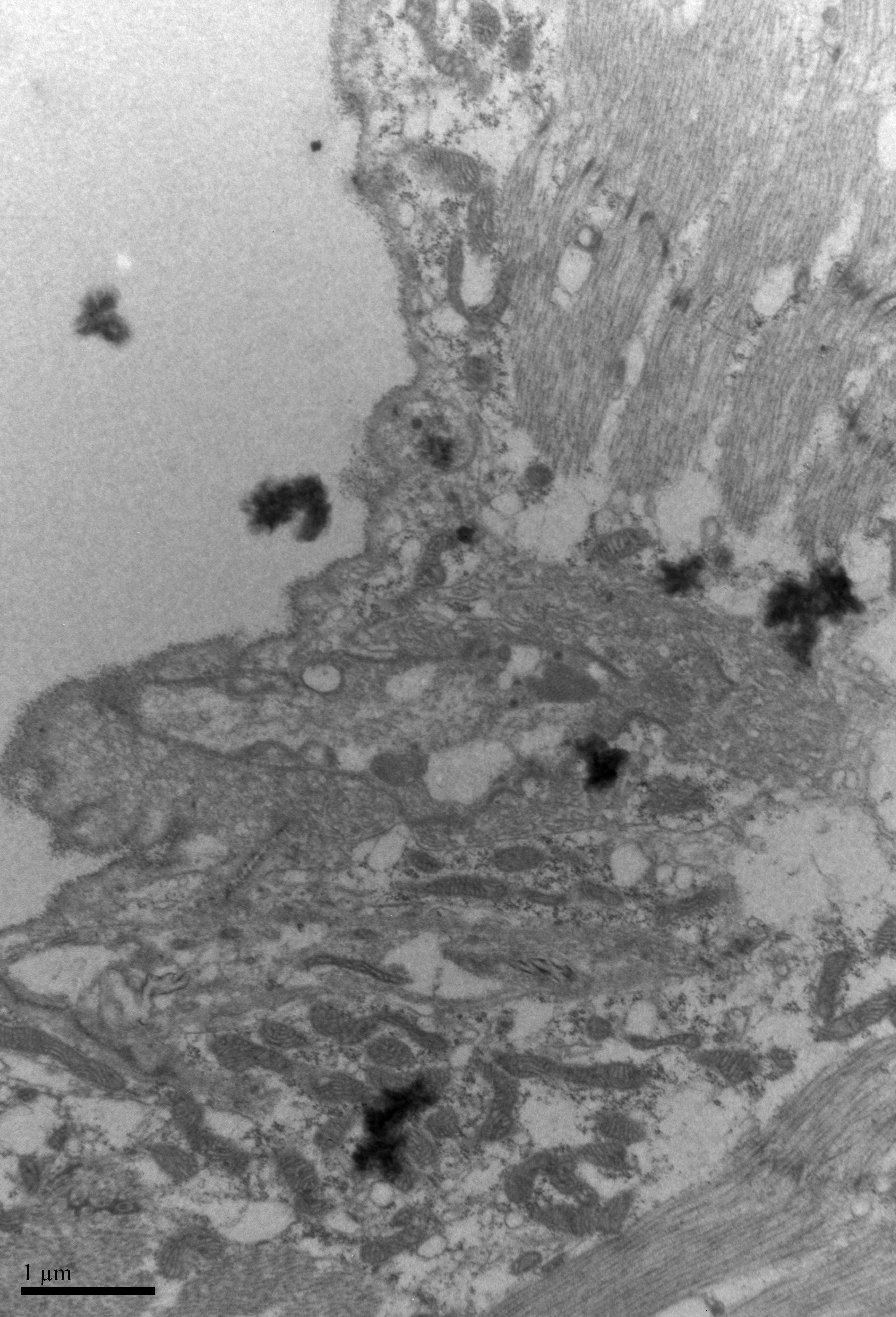

Supplement: Figure 3—source data 1. [file elife-46421-fig3-data1.zip › EM_NPRRdTK/003D-01B-bx2A5-023.jpg]
