# Supplementary material for: Imaging neuropeptide release at synapses with a genetically engineered reporter
Source: eLife. 2019 Jun 26;8:e46421. doi: 10.7554/eLife.46421 (PMC6609332; doi:10.7554/eLife.46421)
Supplement: Supplementary file 2. — Stereological labeling estimations of NPRRANP-GFP and NPRRdTK-GFP, respectively, in Type Ib neurons, or in Type Ib and Type III neurons. Biological controls and internal controls are described in Materials and methods. SNR: Signal-to-Noise Ratio. [file elife-46421-supp2.docx]

|  | **NPRR^ANP-GFP^ (Ib)** | **NPRR^dTK-GFP^** |
| --- | --- | --- |
| average gold particles per DCV | 0.71 | 1.11 |
| gold within DCV area [µm^-2^] | 90.99 | 141.65 |
| gold within bouton area [µm^-2^] | 5.56 | 6.38 |
| gold outside bouton area [µm^-2^] | 1.11 | 0.65 |
|  |  |  |
| **controls** |  |  |
| gold per imaged area [µm^-2^] | 2.18 | 2.47 |
| background (internal control) [µm^-2^] | 0.57 | 0.52 |
| background (biological control) [µm^-2^] | 0.25 | - |
| SNR (gold/DCV area vs. background) | 159.6 | 272.4 |

**Supplementary Table 2:** **Stereological labeling estimates**
