# Supplementary material for: Imaging neuropeptide release at synapses with a genetically engineered reporter
Source: eLife. 2019 Jun 26;8:e46421. doi: 10.7554/eLife.46421 (PMC6609332; doi:10.7554/eLife.46421)
Supplement: Supplementary file 3. — Summary of complete genotypes of transgenic flies used in this study. [file elife-46421-supp3.docx]

**Supplementary Table 3:** **Complete genotypes of transgenic flies used in this study.**

| **Figure** | **Genotype** |
| --- | --- |
| Figure 1C | w; +; GMR57C10-Gal4 (attp2)/UAS-GCaMP6s (su(Hw)attp1) |
| Figure 1C | w; +; GMR57C10-Gal4 (attp2)/UAS-NPRR^ANP^ (attp2) |
| Figure 1D | w; +; GMR57C10-Gal4 (attp2)/UAS-NPTT^ANP-GFP^ (attp2) |
| Figure 2A | w; +; GMR20C11-Gal4 (attp2)/UAS-NPRR^ANP^ (attp2) |
| Figure 2B | w; +; GMR20C11-Gal4 (attp2)/UAS-GCaMP6s (su(Hw)attp1) |
| Figure 2F_1_ | w; UAS-TNT; GMR20C11-Gal4 (attp2)/UAS-NPRR^ANP^ (attp2) |
| Figure 2F_2_ | w; UAS-TNT^imp^; GMR20C11-Gal4 (attp2)/UAS-NPRR^ANP^ (attp2) |
| Figure 3A | w; +; GMR57C10-Gal4 (attp2)/UAS-NPRR^dTK^ (attp2) |
| Figure 3B | w; +; GMR57C10-Gal4 (attp2)/UAS-NPRR^dTK-GFP^ (attp2) |
| Figure 3D | w; +; GMR57C10-Gal4 (attp2)/UAS-NPRR^dTK^ (attp2) |
| Figure 4B | w; +; GMR20C11-Gal4 (attp2)/UAS-NPRR^dTK^ (attp2) |
| Figure 4C | w; +; GMR20C11-Gal4 (attp2)/ UAS-GCaMP6s (su(Hw)attp1) |
| Figure 4D | w; +; GMR19H07-Gal4 (attp2)/UAS-NPRR^ANP^ (attp2) |
| Figure 4E | w; +; GMR19H07-Gal4 (attp2)/UAS-NPRR^dTK^ (attp2) |
| Figure 4F | w; +; GMR19H07-Gal4 (attp2)/ UAS-GCaMP6s (su(Hw)attp1) |
| Figure 1—figure supplement 2A | w; +; GMR57C10-Gal4 (attp2)/UAS-mCD8::GFP (attp2) |
| Figure 1—figure supplement 2B | w; +; GMR57C10-Gal4 (attp2)/UAS-GCaMP6s (su(Hw)attp1) |
| Figure 1—figure supplement 2C | w; +; GMR57C10-Gal4 (attp2)/UAS-NPRR^ANP^ (attp2) |
| Figure 1—figure supplement 3A | w; +; GMR20C11-Gal4 (attp2)/UAS-GCaMP6s (su(Hw)attp1) |
| Figure 1—figure supplement 3B | w; +; GMR20C11-Gal4 (attp2)/UAS-mCD8::GFP (attp2) |
| Figure 1—figure supplement 3C | w; +; GMR20C11-Gal4 (attp2)/UAS-NPRR^dTK^ (attp2) |
| Figure 1—figure supplement 3D | w; +; GMR20C11-Gal4 (attp2)/UAS-NPRR^ANP^ (attp2) |
| Figure 1—figure supplement 4A | w; +; GMR57C10-Gal4 (attp2)/UAS-NPRR^ANP^ (attp2) |
| Figure 1—figure supplement 4B | w; +; GMR57C10-Gal4 (attp2)/UAS-NPRR^ANP-GFP^ (attp2) |
| Figure 2—figure supplement 1 | w; +; GMR20C11-Gal4 (attp2)/UAS-NPRR^ANP^ (attp2) |
| Figure 2—figure supplement 2C_1_ | w; UAS-TNT^imp^; GMR20C11-Gal4 (attp2)/UAS-GCaMP6s (su(Hw)attp1) |
| Figure 2—figure supplement 2C_2_ | w; UAS-TNT; GMR20C11-Gal4 (attp2)/UAS-GCaMP6s (su(Hw)attp1) |
| Figure 4—figure supplement 1 | w; +; GMR20C11-Gal4 (attp2)/UAS-NPRR^ANP^ (attp2) |
| Figure 4—figure supplement 1 | w; +; GMR20C11-Gal4 (attp2)/UAS-NPRR^dTK^ (attp2) |
